# Relationship between interaction geometry and cooperativity measured in H-bonded networks of hydroxyl groups†

**DOI:** 10.1039/d5sc00784d

**Published:** 2025-03-27

**Authors:** Lucia Trevisan, Andrew D. Bond, Christopher A. Hunter

**Affiliations:** a Yusuf Hamied Department of Chemistry, University of Cambridge Lensfield Road Cambridge CB2 1EW UK herchelsmith.orgchem@ch.cam.ac.uk

## Abstract

Cooperativity between interactions in H-bonded networks can increase the strengths of H-bonds involving hydroxyl groups by up to 50%. The effect of changing the geometry of an intramolecular hydroxyl–hydroxyl H-bond on cooperativity with an intermolecular hydroxyl·quinuclidine H-bond was quantified by comparing the H-bonding properties of a series of hydroxycresols with the corresponding series of bisphenols. In the hydroxycresols, the intramolecular H-bond forms a 6-membered ring, and X-ray crystallography showed that the H-bond is distorted away from the ideal linear O–H⋯O geometry by up to 35°. In the bisphenols, the intramolecular H-bond forms an 8-membered ring, and the geometry is close to ideal, with the OH bond of the donor pointing directly at the lone pair of the acceptor. The presence of the intramolecular H-bonding interactions in solution was confirmed using ^1^H NMR spectroscopy, and NMR titrations were used to measure the association constants for formation 1 : 1 complexes with quinuclidine in *n*-octane. Compared with the non-cooperative H-bond formed by benzyl alcohol with quinuclidine, the strength of the intermolecular H-bond formed by the hydroxycresols increased by between −8 kJ mol^−1^ and −14 kJ mol^−1^, depending on the substituent *para* to the phenol OH donor. Electron-withdrawing substituents make the phenol a better H-bond donor, and the increase in the strength of the intramolecular H-bond leads to an increase in the strength of the intermolecular H-bond with quinuclidine. For the bisphenols, the cooperative effects were very similar: the substituent effects were practically identical, and the presence of the intramolecular H-bond increased the strength of the intermolecular interaction by between −10 kJ mol^−1^ and −16 kJ mol^−1^. The results show that cooperativity in H-bonded networks depends strongly on the polarity of the interacting groups but is relatively insensitive to the precise geometric arrangement.

## Abstract 2

Cooperativity in H-bonded networks of hydroxyl groups depends strongly on the polarity of the interacting groups but is relatively insensitive to the precise geometric arrangement and alignment of the OH bonds.

## Graphical abstract

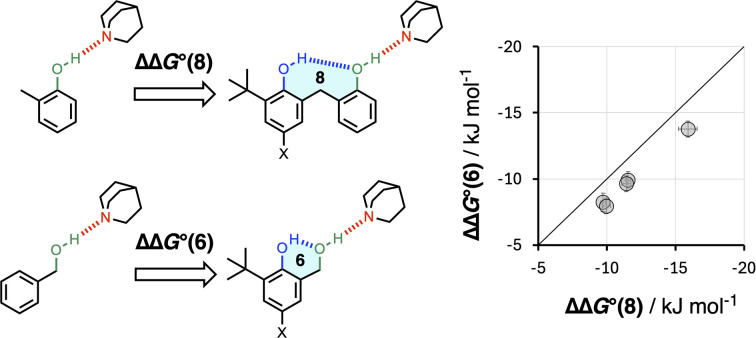

## Introduction

Hydrogen bonds are one of the most important non-covalent interactions that determine the properties of biomolecules^1–3^ and synthetic supramolecular systems.^4–7^ In networks of H-bonding interactions, cooperativity changes the properties of the interactions compared to isolated systems that form a single H-bond.^8–12^ H-bond cooperativity for alcohols was first observed using infrared spectroscopy, and it was shown that when the alcohol hydroxyl group acts as a H-bond donor in the interaction with a H-bond acceptor, the strength of a second H-bonding interaction in which the hydroxyl group acts as a H-bond acceptor increases.^13–15^ Experiments on molecular torsion balances showed that the free energy change associated with interaction of a formamide H-bond acceptor with a hydroxyl group becomes more favourable when the hydroxyl group also acts as an acceptor in a second intramolecular H-bond.^16–18^

**Fig. 1:**
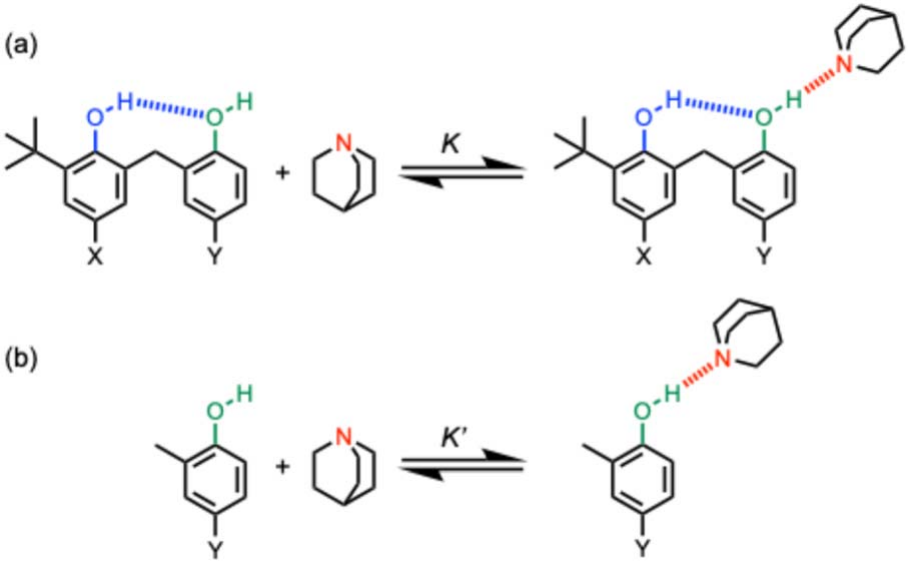
(a) Complexes used to quantify cooperativity in H-bond networks by measuring the effect of an intramolecular H-bond between a phenol donor (blue) and a phenol acceptor (green) on the interaction with quinuclidine (red). The X and Y substituents tune the H-bond properties of the blue and green hydroxyl groups respectively. (b) Reference interaction of quinuclidine with phenols that do not make an intramolecular H-bond.

We have measured the effect of an intramolecular H-bond in which a hydroxyl group acts as a H-bond acceptor on the strength of a second intermolecular H-bond in which the hydroxyl group acts as a H-bond donor, and again positive cooperativity was observed.^19^ The approach is shown in Fig. 1. The intramolecular hydroxyl–hydroxyl H-bond shown in blue in Fig. 1a modifies the properties of the alcohol H-bond donor in green, and the strength of this intramolecular H-bond can be tuned by using different X and Y substituents.^20^ Comparison of the strength of the intermolecular H-bond of the green hydroxyl group with quinuclidine (*K* in Fig. 1a) with the corresponding interaction of quinuclidine with a phenol that does not make an intramolecular H-bond (*K*′ in Fig. 1b) can be used to quantify the cooperativity between the intramolecular and intermolecular H-bonds in the H-bonded network in Fig. 1a. By changing the X substituent, it was found that the cooperative enhancement of the intermolecular interaction with quinuclidine was directly proportional to the strength of the intramolecular H-bond. By changing the Y substituent, it was found that the cooperative enhancement of the intermolecular interaction with quinuclidine was independent of the properties of the green hydroxyl group.^20^ Here we investigate the effect of changing the geometry of the intramolecular H-bond on the magnitude of the cooperative effects in H-bonded networks.

**Fig. 2:**
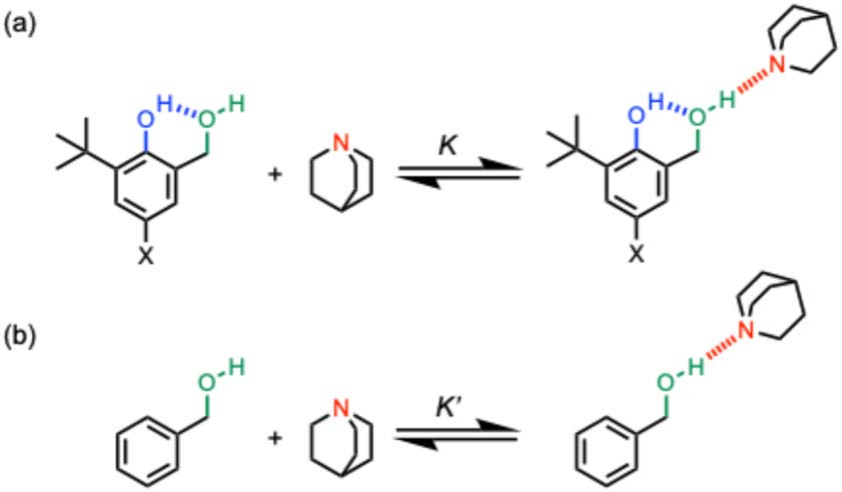
(a) Complexes used to quantify cooperativity in H-bond networks by measuring the effect of an intramolecular H-bond between a phenol donor (blue) and a benzyl alcohol acceptor (green) on the interaction with quinuclidine (red). The X substituent is used to tune the strength of the blue intramolecular H-bond in the hydroxylcresol derivatives. (b) Reference interaction of quinuclidine with a benzyl alcohol that does not make an intramolecular H-bond.

The approach shown in Fig. 2 is very similar to that in Fig. 1, except that the green hydroxyl group is moved closer to the blue hydroxyl group, and as a result, the size of the H-bonded ring is reduced from 8 atoms to 6 atoms. Comparison of the strength of the intermolecular H-bond of the green hydroxyl group with quinuclidine (*K* in Fig. 2a) with the corresponding interaction of quinuclidine with a benzyl alcohol that does not make an intramolecular H-bond (*K*′ in Fig. 2b) can be used to quantify the cooperativity between the intramolecular and intermolecular H-bonds in the H-bonded network in Fig. 2a. The strength of the intramolecular H-bond can be tuned by using different X substituents, and comparison of the ratios *K*/*K*′ for the hydroxycresol system in Fig. 2a with the results for the corresponding bisphenol system in Fig. 1a provide a quantitative measurement of the effect of H-bond geometry on cooperativity. We note that changing the green hydroxyl group from a phenol to a benzyl alcohol could also contribute to any differences observed, but we have previously shown that changing the polarity of the green hydroxyl group has no effect on cooperativity in the bisphenol system,^20^ so this effect is likely to be small.

## Results

**Fig. 3:**
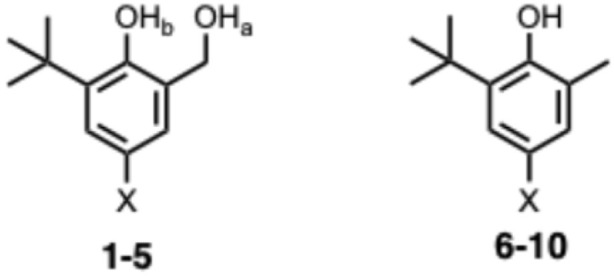
Chemical structures of hydroxycresols (1 X = NO_2_, 2 X = Br, 3 X = F, 4 X = Me, 5 X = NMe_2_) and reference phenols (6 X = NO_2_, 7 X = Br, 8 X = F, 9 X = Me, 10 X = NMe_2_). The ^1^H NMR labelling scheme for the hydroxyl groups is shown.

The compounds used to carry out the experiment in Fig. 2 are shown in Fig. 3. Hydroxycresols 1–5 are equipped with a series of different substituents (X = NO_2_, Br, F, CH_3_ and NMe_2_) to tune the strength of the intramolecular H-bond. Compounds 6–10 are the corresponding reference compounds that quantify the substituent effects on the H-bond donor properties of the blue hydroxyl group in the absence of the intramolecular H-bond. Compound 9 was commercially available, the synthesis of compound 5 is reported in the ESI,† and all of the other compounds in Fig. 3 were synthesised as described previously.^20^

### Intramolecular H-bonding interactions

**Fig. 4:**
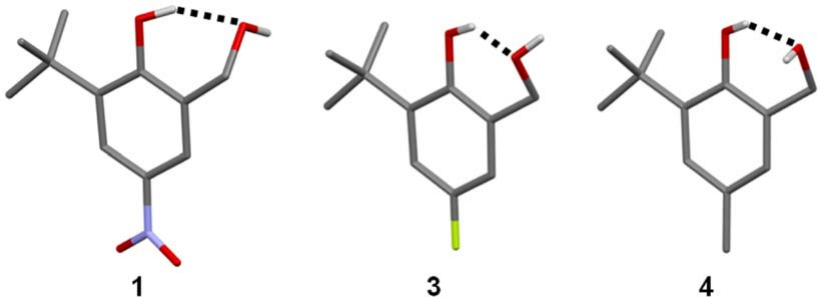
Molecular structures taken from the X-ray crystal structures of 1, 3 and 4.^21^ Intramolecular H-bonding interactions are shown as dotted lines.

Crystals suitable for X-ray diffraction were obtained for compounds 1 and 3, while the crystal structure of 4 has been reported previously.^21^ For 1, the H atoms of the OH groups were located and refined using the X-ray data, showing unambiguously the existence of the intramolecular H-bond involving the phenolic hydroxyl group OH_b_ as the donor and the benzylic hydroxyl group OH_a_ as the acceptor (Fig. 4). For 3 and 4, the H atom positions were not established directly from the X-ray data, but the presence of the intramolecular H-bond can be inferred from the fact that OH_a_ is the only H-bond acceptor in the vicinity of OH_b_. All three structures show minimal deviation when subjected to geometry optimization using periodic dispersion-corrected DFT calculations, which adds confidence to the validity of the H-atom positions.^22^

**Fig. 5:**
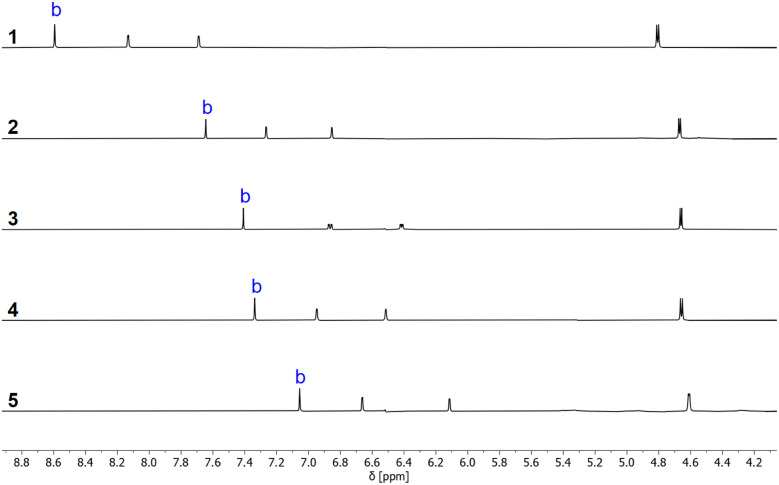
Partial ^1^H-NMR spectra of 0.24 mM solutions of hydroxycresols 1–5 recorded at 298 K in *n*-octane with WET solvent suppression. The signal due to the phenol OH is labelled with b.

^1^H NMR spectra recorded in deuterochloroform indicate that the intramolecular H-bonding interactions present in the solid state persist in the solution. It was possible to use two-dimensional NMR experiments (COSY, HSQC and HMBC) to assign the OH signals for all of compounds 1–5, and Table 1 reports the ^1^H-NMR chemical shifts of the signal due to the phenolic proton OH_b_. The values are all more than 3 ppm higher than the chemical shift of the OH signal for the corresponding reference phenols 6–10, which do not make an intramolecular H-bond. The signal due to OH_a_ appears as a triplet between 2.0 and 2.5 ppm for all of compounds 1–5, which is only slightly higher in chemical shift than the OH signal for benzyl alcohol (1.7 ppm).^23^ These results show that there is an intramolecular H-bond in compounds 1–5 in solution and that it is OH_b_ that acts as the H-bond donor.^24,25^^1^H-NMR spectra were also recorded in *n*-octane using WET solvent suppression.^26^ For compounds 1–5, the singlet labelled b in Fig. 5 could be assigned to the phenolic proton OH_b_, because the chemical shift is very similar to the value measured in deuterochloroform. The signal due to OH_a_ was not visible in the *n*-octane spectra, because it was too close to the solvent signals that are suppressed.

**Table 1:** ^1^H NMR chemical shifts of the signals due to phenol OH protons measured in deuterochloroform at 298 K

X	Hydroxycresol	*δ*(OH_b_)/ppm	Phenol^20^	*δ*(OH)/ppm
NO_2_	1	8.9	6	5.5
Br	2	7.8	7	4.7
F	3	7.6	8	4.5
CH_3_	4	7.5	9	4.6
NMe_2_	5	7.2	10	4.3

### Intermolecular H-bonding interactions

**Fig. 6:**
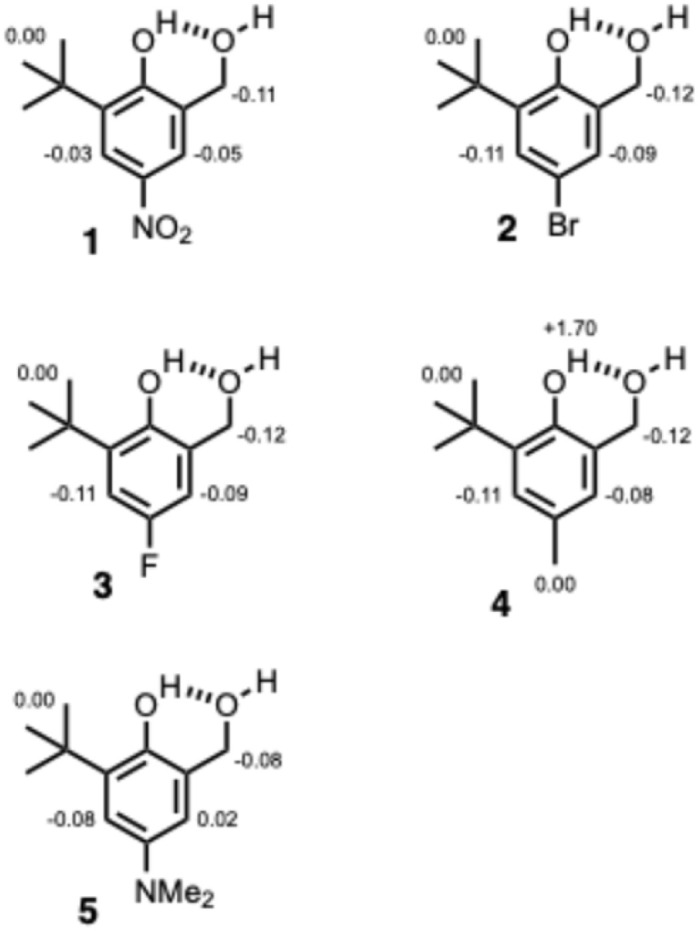
Limiting complexation-induced changes in ^1^H-NMR chemical shift (ppm) for the formation of 1 : 1 complexes of 1–5 with quinuclidine in *n*-octane at 298 K.

The formation of intermolecular H-bonds with quinuclidine in *n*-octane was investigated using ^1^H NMR and UV-vis absorption titrations. For compounds 1–4, the UV-Vis titration data fit well to a 1 : 1 binding isotherm, and the resulting association constants are reported in Table 2 (see ESI† for details). For compound 5 and the reference compound, benzyl alcohol, the changes in the UV-vis spectrum were too small to obtain reliable association constants, so the interaction with quinuclidine was quantified using ^1^H NMR titrations. For compounds where association constants could be determined by both ^1^H NMR and UV-Vis absorption titrations, the results were consistent.^19^^1^H NMR dilution experiments in *n*-octane showed that there is no self-association at millimolar concentrations for any of compounds 1–5. The changes in ^1^H NMR chemical shift observed on addition of quinuclidine fit well to a 1 : 1 binding isotherm, and the limiting complexation-induced changes in chemical shift are shown in Fig. 6 (see ESI† for details). When quinuclidine was added to compounds 1–5, the signal due to the phenol OH proton became too broad to detect for all but compound 4, and as explained above the signal due to OH_a_ was not observed in *n*-octane due to the solvent suppression.

**Table 2:** Association constants for formation of 1 : 1 complexes with quinuclidine in *n*-octane at 298 K^a^

X	Hydroxycresol	*K*_a_/M^−1^	Phenol^b^	*K*_a_/M^−1^
NO_2_	1	4400 ± 300	6	490 ± 20
Br	2	930 ± 90	7	93 ± 14
F	3	830 ± 40	8	78 ± 9
CH_3_	4	470 ± 70	9	20 ± 6
NMe_2_	5	420 ± 10	10	13 ± 4

a Errors are the standard error of the mean of three independent UV-Vis absorption or ^1^H NMR titrations.

b These values were reported previously.^20^

Although it is not possible to assign directly the site of interaction with quinuclidine, the evidence points strongly to a H-bond with the benzylic hydroxyl group OH_a_. The crystal structures and solution phase NMR experiments all indicate that there is an intramolecular H-bond in which OH_b_ acts as the donor and OH_a_ acts as the acceptor in compounds 1–5. The largest complexation-induced change in ^1^H NMR chemical shift on binding quinuclidine was observed for the benzylic CH_2_ proton in all cases, and no change in chemical shift was observed for the *t*-butyl signal, which suggests that quinuclidine sits closer to OH_a_ than OH_b_ in the complex. The large increase in chemical shift observed for OH_b_ in compound 4 is consistent with the changes in chemical shift that we have observed previously in chains of H-bonded hydroxyl groups:^19^ formation of the intermolecular H-bond between OH_a_ and quinuclidine increases the strength of the intramolecular H-bond and causes a corresponding increase in the chemical shift of OH_b_. The association constants for formation of 1 : 1 complexes with compounds 1–5 are all an order of magnitude higher than the values measured for the corresponding reference phenols 6–10, which is consistent with an interaction between quinuclidine and OH_a_ for compounds 1–5. If OH_b_ were involved in the intermolecular interaction with quinuclidine for compounds 1–5, then the intramolecular H-bond with OH_b_ would first have to be broken, which would lead to a decrease in association constant compared with compounds 6–10 rather than the increase that is observed.

The association constant measured for formation of a 1 : 1 complex between benzyl alcohol and quinuclidine is 17 ± 4 M^−1^, which is 1–2 orders of magnitude lower than the values measured for compounds 1–5. This result indicates significant cooperativity between formation of the intramolecular and intermolecular H-bonds. The strength of the intramolecular H-bond depends on the *para* substituent on the phenol ring, and the results in Table 2 show a clear correlation between the strengths of the intramolecular and intermolecular H-bonding interactions. For example, the most electron withdrawing substituent, NO_2_, leads to a large increase in the H-bond donor strength of the phenol (*cf.* compound 6), and there is a correspondingly large increase in the strength of the intermolecular H-bond formed by compound 1 with quinuclidine.

Table 3 compares the cooperativity factors (*K*/*K*′) measured for the hydroxylcresol derivatives in this work (Fig. 2) with the values measured previously for the analogous bisphenols shown in Fig. 1 with Y = Me.^20^ The cooperativity factors in the hydroxycresols are a factor of two lower than the values measured for the bisphenols, but the substituent effects are very similar in the two different systems.

**Table 3:** Cooperative enhancement of intermolecular H-bonding interactions with quinuclidine measured in *n*-octane at 298 K (*K*/*K*′)^a^

X	Hydroxycresol	Bisphenol (Y = Me)^20^
NO_2_	259 ± 63	611 ± 170
Br	55 ± 14	106 ± 8
F	49 ± 12	100 ± 12
CH_3_	28 ± 8	51 ± 3
NMe_2_	25 ± 6	56 ± 6

a Errors are the standard error of the mean of three independent experiments.

## Discussion

**Fig. 7:**
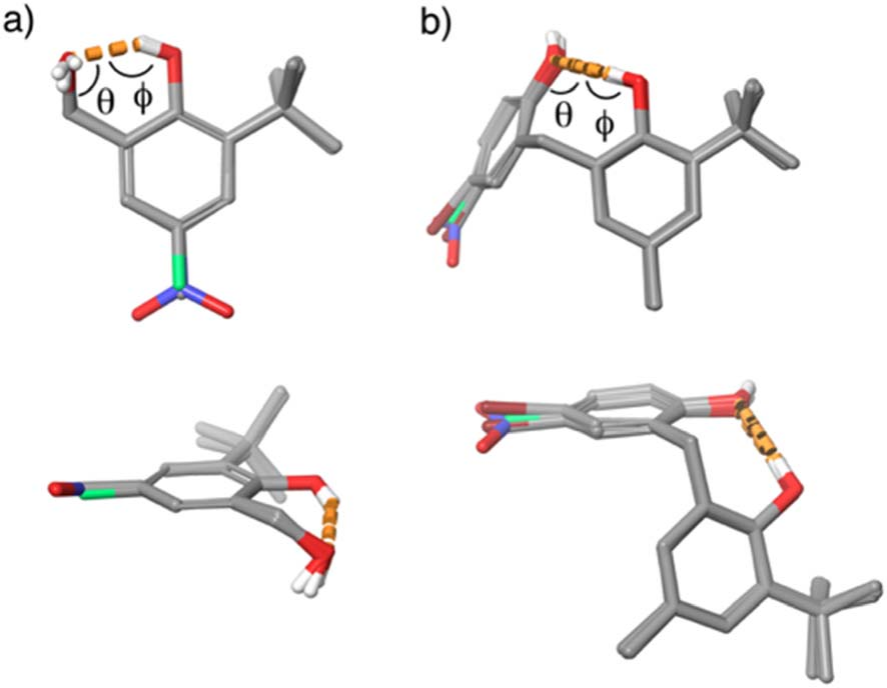
(a) Two views of the overlaid molecular structures of the hydroxycresols 1, 3 and 4 taken from the corresponding crystal structures. (b) Two views of the overlaid molecular structures of the bisphenols in Fig. 1a (X = Me, Y = NO_2_, Br, F and Me) taken from the corresponding crystal structures. In each case, the overlays were obtained by matching the heavy atoms of the phenol rings and the H-bonded rings. Intramolecular H-bonding interactions are shown as orange dotted lines.

The crystal structures of the hydroxycresol and bisphenol derivatives were used to quantify the change in the interaction geometry for the intramolecular H-bonds between the 6-membered ring and the 8-membered ring. Fig. 7a shows an overlay of the molecular structures of 1, 3 and 4. The conformations are almost identical, and the geometry of the 6-membered H-bonded ring is very similar in all cases. Table 4 compares the angles and distances that describe the geometry of the intramolecular H-bond, using H-atom positions taken from DFT-optimised crystal structures. The vector of the OH bond of the H-bond donor (OH_b_) does not point directly at the oxygen of the H-bond acceptor: *ϕ* is 145–151° rather than 180°. The value of *θ* shows that the hydrogen of the H-bond donor (OH_b_) does not sit on the lone pair direction of the oxygen of the H-bond acceptor: *θ* is 87–94° rather than 109°. Thus the constraints of the intramolecular interaction in the 6-membered ring significantly distort the H-bond away from the ideal geometry.

**Table 4:** Geometrical parameters for intramolecular H-bonds in X-ray crystal structures of hydroxycresols 1, 3 and 4^a^

X	*d*(O⋯O)/Å	*d*(O–H)/Å	*d*(H⋯O)/Å	*ϕ*/deg	*θ*/deg
NO_2_	2.620	1.01	1.69	151.3	90.6
F	2.736	0.99	1.85	147.7	86.9
F	2.644	1.00	1.75	147.4	91.6
F	2.706	0.99	1.83	145.5	89.5
Me	2.617	0.99	1.73	146.9	93.6
Me	2.685	0.99	1.81	144.5	93.0

a H-atom positions are taken from DFT-optimised crystal structures. Duplicate entries refer to independent molecules in the asymmetric unit. See Fig. 7a for the definition of *ϕ* and *θ*.

Fig. 7b and Table 5 show the corresponding results for the 8-membered H-bonded ring in the crystal structures of four different bisphenol derivatives. Again the conformations are almost identical, and the geometry of the intramolecular H-bond is very similar in all cases. For the 8-membered ring, the geometry of the H-bond is close to ideal: the vector of the OH bond of the donor points directly at the oxygen of the acceptor (*ϕ* is 167–178°), and the hydrogen of the donor sits close to the lone pair direction of the acceptor (*θ* is 115–119°). It is clear that the geometries of the intramolecular H-bonds are quite different in the 6-membered and 8-membered rings, and that the 6-membered ring distorts the interaction much further from an idealized arrangement.

**Table 5:** Geometrical parameters for intramolecular H-bonds in X-ray crystal structures of bisphenols (X = Me)^a^

Y	*d*(O⋯O)/Å	*d*(O–H)/Å	*d*(H⋯O)/Å	*ϕ*/deg	*θ*/deg
NO_2_	2.789	0.99	1.82	167.2	115.7
F	2.688	1.00	1.68	177.9	116.3
F	2.720	1.00	1.72	176.8	118.0
Me	2.738	1.00	1.74	175.7	114.9
Me	2.717	1.00	1.72	173.3	118.6
Br	2.715	1.00	1.72	173.5	118.6
Br	2.736	1.00	1.74	174.9	116.4

a H-atom positions are taken from DFT-optimised crystal structures. Duplicate entries refer to independent molecules in the asymmetric unit. See Fig. 7b for the definition of *ϕ* and *θ*.

Although it is possible that the molecules adopt a wider range of conformations in solution compared with the crystal structures in Fig. 7, the fact that the geometries of the intramolecular H-bonds are conserved in the crystal structures of different compounds gives some confidence that these structures are a feature of well-defined intramolecular interactions rather than something imposed by crystal packing. Moreover, conformational searches using molecular mechanics calculations (OPLS 2005 with chloroform solvation) returned the same structures as the lowest energy solution-phase conformations.^27^

**Fig. 8:**
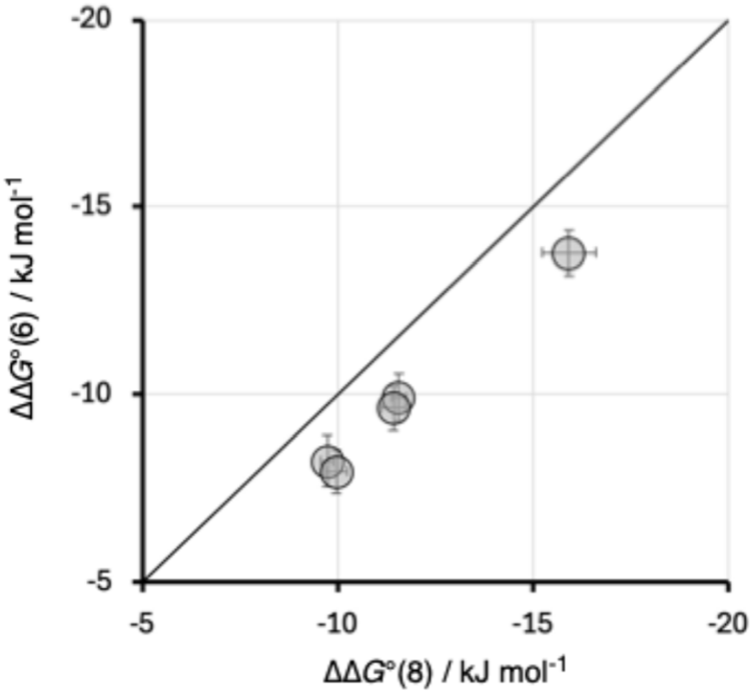
Relationship between cooperative enhancement of intermolecular H-bonding interactions in compounds with 6-membered and 8-membered intramolecular H-bonded rings (ΔΔ*G*°(6) and ΔΔ*G*°(8)). The line is *y* = *x*.

The cooperativity enhancement factors in Table 3 can be used to calculate the change in free energy of the intermolecular interaction with quinuclidine due to the presence of the intramolecular H-bond (ΔΔ*G*° = −*RT* ln(*K*/*K*′)). Fig. 8 compares the values of ΔΔ*G*°(6) for the hydroxycresol compounds, which have the 6-membered H-bonded ring, with the values obtained for the 8-membered H-bonded ring in the corresponding bisphenols with the same X substituent, ΔΔ*G*°(8). Despite the difference between the geometries of the types of intramolecular H-bond, the magnitude of the cooperativity observed is very similar, and the substituent effects are practically identical. Cooperativity associated with the 6-membered H-bonded ring is worth about 2 kJ mol^−1^ less than in the 8-membered H-bonded ring, but this difference appears to be a constant, which is independent of the X substituent and strength of the intramolecular H-bond. We conclude that cooperative effects are relatively insensitive to changes in interaction geometry, and distortions of up to 35° away from the ideal linear O–H⋯O alignment have little effect.

## Conclusions

The effect of changing the geometry of an intramolecular hydroxyl–hydroxyl H-bond on cooperativity with an intermolecular hydroxyl·quinuclidine H-bond was quantified by comparing the H-bonding properties of a series of hydroxycresols with the corresponding series of bisphenols. In the hydroxycresols, the intramolecular H-bond forms a 6-membered ring, and X-ray crystallography showed that the H-bond is distorted away from the ideal linear O–H⋯O geometry by up to 35°. In the bisphenols, the intramolecular H-bond forms an 8-membered ring, and the geometry is close to ideal, with the OH bond of the donor pointing directly at the lone pair of the acceptor. A series of compounds were compared in which the substituent *para* to the hydroxyl group of the phenol H-bond donor was used to tune the strength of the intramolecular interaction, and hence quantify the relationship with the strength of the intermolecular interaction of the other hydroxyl group with quinuclidine.

X-ray crystal structures of three of the hydroxycresol derivatives showed that the phenol OH group acts as the H-bond donor and the benzyl OH group acts as the acceptor in the intramolecular interaction, and ^1^H NMR spectroscopy confirmed that this interaction persists in solution in both chloroform and in *n*-octane for all of the compounds. NMR titrations were used to measure the association constants for formation of 1 : 1 complexes with quinuclidine. The complexation-induced changes in chemical shift indicate that the intramolecular H-bond is intact in the complex, and the benzyl hydroxyl group acts as the H-bond donor in the interaction with quinuclidine. The association constants measured for the hydroxylcresols are all orders of magnitude larger than the association constant for the benzyl alcohol·quinuclidine complex in which there is no intramolecular H-bond. The magnitude of the cooperative enhancement of the intermolecular H-bond depends on the substituent *para* to the phenol that acts as the H-bond donor in the intramolecular interaction. Electron-withdrawing substituents that increase the strength of the intramolecular H-bond lead to a larger increase in strength of the intermolecular H-bond (−14 kJ mol^−1^ for NO_2_) compared with electron-donating groups (−8 kJ mol^−1^ for NMe_2_).

The results for the hydroxycresol derivatives are very similar to those obtained for the corresponding series of bisphenols.^20^ The magnitude of the cooperative effects measured in the bisphenols is about 2 kJ mol^−1^ larger than in the hydroxycresols, but the substituent effects are practically identical. The results indicate that the difference in the geometries of the intramolecular H-bonds in the 6-membered and 8-membered rings does not have a significant effect on the magnitude of the cooperative effects. We conclude that the very large cooperative effects that we have measured in networks of hydroxyl–hydroxyl H-bonds depend strongly on the polarity of the interacting groups but are relatively insensitive to the precise geometric arrangement.

## Data availability

All supporting data is provided in the ESI.†

## Author contributions

The manuscript was written through contributions of all authors.

## Conflicts of interest

There are no conflicts to declare.

## Supplementary Material

SC-OLF-D5SC00784D-s001

SC-OLF-D5SC00784D-s002
