# Supplementary material for: Relationship between interaction geometry and cooperativity measured in H-bonded networks of hydroxyl groups
Source: Chem Sci. 2025 Mar 27;16(17):7418–23. doi: 10.1039/d5sc00784d (PMC11947983; doi:10.1039/d5sc00784d)
Supplement: SC-016-D5SC00784D-s001 [file SC-016-D5SC00784D-s001.pdf]

Supplementary Information (SI) for Chemical Science.
This journal is © The Royal Society of Chemistry 2025

Relationship between Interaction Geometry and Cooperativity Measured in H-Bonded Networks of Hydroxyl Groups

Lucia Trevisan, Andrew D. Bond and Christopher A. Hunter*

[a] Yusuf Hamied Department of Chemistry, University of Cambridge, Lensfield Road, Cambridge, CB2 1 EW, UK.

Supporting Information

Table of contents	Page
**Materials and Methods**	S2
**Synthesis**	S3
**NMR Characterisation**	S5
**Single-Crystal X-ray Crystallography**	S12
Geometry optimisation using dispersion-correct DFT calculations	S15
**NMR Experiments**	S16
^{1}H-NMR Titration – General Procedure	S16
^{1}H-NMR Dilution – General Procedure	S17
Molecule 1	S18
Molecule 2	S19
Molecule 3	S20
Molecule 4	S21
Molecule 5	S22
Benzyl alcohol	S26
NOESY Experiments	S30
**UV-Vis Experiments**	S35
UV-Vis Titration – General Procedure	S35
UV-Vis Dilution – General Procedure	S35
Molecule 1	S37
Molecule 2	S39
Molecule 3	S41
Molecule 4	S43
**References**	S45

1. Materials and Methods

All reagents were purchased from commercial sources (Sigma Aldrich UK, Acros, Fluorochem) and were used as received without any further purification. Dry solvents were obtained by means of a Grubbs solvent system.

Flash chromatography was done with an automated system (Combiflash Companion) using pre-packed cartridges of silica (50 µm PuriFlash® column) or reverse phase C18HP (15 µm, PuriFlash® column).

The LC-MS analysis of samples was performed using Waters Acquity H-class UPLC coupled with a single quadrupole Waters SQD2. ACQUITY UPLC CSH C18 Column, 130 Å, 1.7 µm, 2.1 mm X 50 mm was used as the UPLC column for all samples. The conditions of the UPLC method are as follows: Solvent A: Water +0.1% Formic acid; Solvent B: Acetonitrile +0.1% Formic acid; Gradient of 0-2 minutes 5% -100%B + 1 minute 100% B with re-equilibration time of 2 minutes. Flow rate: 0.6 ml/min; column temperature of 40°C; injection volume of 2 µL. The signal was monitored with MS-ES$^+$, MS ES$^-$, at 254nm or at 290 nm.

^{1}H-NMR and ^{13}C-NMR were recorded on a 400 MHz, 500 MHz, 600 MHz or 700 MHz Bruker spectrometer as indicated. The reference values used for the chemical shifts of the various spectra are reported in the literature.[1] The splitting pattern is indicated with the following abbreviations: s for singlet, br s for broad singlet, d for doublet, t for triplet, q for quartet, quint for quintet, m for multiplet, dd for doublet of doublets and dt for doublet of triplets.

FT-IR spectra were collected with an ALPHA FT-IR Spectrometer from Bruker.

UV-Vis spectra were recorded with a UV-Vis Cary 60 spectrophotometer (Agilent).

Melting points were recorded with a Mettler Toledo MP90 melting point apparatus.

2. Synthesis

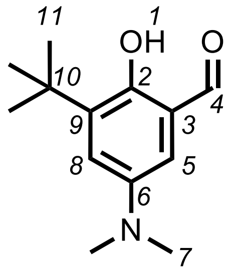

17

Modified from a previously reported procedure.[2]

To a solution of 3-(*tert*-butyl)-2-hydroxy-5-nitrobenzaldehyde (1.0996 g, 4.9 mmol) and a 37% wt aqueous solution of formaldehyde (26.0 mL, 349.2 mmol) in ethanol (80 mL), palladium on activated charcoal (10% wt, 656.6 mg, 0.62 mmol) was added followed by more ethanol (20 mL). The reaction mixture was stirred under 1 bar of hydrogen at room temperature for 24 hours. Then the Pd/C was removed by filtration on Celite and the volume of the filtrate was reduced by half under reduced pressure. Water was added to the solution resulting in the formation of a precipitate that was washed with water (250 mL). The product was dried under vacuum and it was obtained as an orange solid (0.8438 g, 3.8 mmol, 77%).

^{1}H-NMR (400 MHz, $CDCl_3$) δ_H (ppm): 11.28 (s, 1H, H(1)), 9.85 (s, 1H, H(4)), 7.15 (d, J = 3.0 Hz, 1H, H(8)), 6.72 (d, J = 3.1 Hz, 1H, H(5)), 2.90 (s, 6H, H(7)), 1.43 (s, 9H, H(11)).

^{13}C{^{1}H}-NMR (101 MHz, $CDCl_3$) δ_C (ppm): 197.5 (1C, C(4)), 154.3 (1C, C(2)), 144.4 (1C, C(6)), 139.0 (1C, C(9)), 122.9 (1C, C(8)), 120.5 (1C, C(3)), 114.8 (1C, C(5)), 42.1 (1C, C(7)), 35.3 (1C, C(10)), 29.4 (3C, C(11)).

HRMS: calc. for $C_{13}H_{20}NO_2^+$ $[M+H]^+$ 222.1494, found 222.1492.

IR spectrum $\tilde{v}$ (cm^{-1}): 2910 ($v_{\text{O-H alcohol}}$), 3065-2996 ($v_{\text{C-H alkene}}$), 2974-2834 ($v_{\text{C-H alkane}}$), 1650 ($v_{\text{C=O aldehyde}}$), 1612 ($v_{\text{C=C cyclic alkene}}$), 1459 ($\delta_{\text{C-H alkane, methyl group}}$), 1355 ($\delta_{\text{O-H phenol}}$), 1059 ($v_{\text{C-N amine}}$).

m.p.: 135.6-136.1°C (lit. 133.7-134°C[3]).

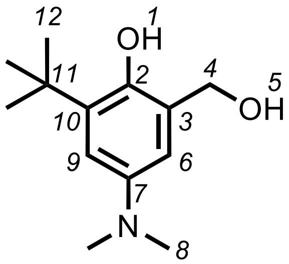

5

To a vigorously stirred solution 3-(*tert*-butyl)-5-(dimethylamino)-2-hydroxybenzaldehyde **17** (632.4 mg, 2.9 mmol) in methanol cooled to 0°C, sodium borohydride (394.2 mg, 10 mmol) was added. The reaction was then warmed to room temperature and stirred for 22.5 hours. The solvent was removed under reduced pressure, glacial acetic acid (6 mL) and water (50 mL) were added. The aqueous phase was extracted with DCM (1x50 mL) and ethyl acetate (4x100 mL). The combined organic phases were dried over magnesium sulphate, filtered and dried under reduced pressure. The crude product was purified by flash column chromatography (SiO_2, 0-10% gradient of methanol in DCM). A light orange, brownish solid was obtained as the product (562.6 mg, 2.5 mmol, 88%).

^{1}H-NMR (600 MHz, $CDCl_3$) δ_H (ppm): 7.24 (s, 1H, H(1)), 6.79 (s, 1H, H(9)), 6.30 (s, 1H, H(6)), 4.78 (s, 2H, H(4)), 2.81 (s, 6H, H(8)), 1.43 (s, 9H, H(12)).

^{13}C{^{1}H}-NMR (151 MHz, $CDCl_3$) δ_C (ppm): 148.1 (1C, C(2)), 144.4 (1C, C(7)), 138.0 (1C, C(10)), 125.4 (1C, C(3)), 114.2 (1C, C(9)), 112.1 (1C, C(6)), 66.1 (1C, C(4)), 42.4 (1C, C(8)), 35.2 (1C, C(11)), 29.8 (3C, C(12)).

HRMS: calc. for $C_{13}H_{22}NO_2^+$ $[M+H]^+$ 224.1651, found 224.1652.

IR spectrum $\tilde{\nu}$ (cm^{-1}): 3282 ($\nu_{O\text{-}H\ alcohol}$), 2994 ($\nu_{C\text{-}H\ alkene}$), 2953-2793 ($\nu_{C\text{-}H\ alkane}$), 1610 ($\nu_{C=C\ cyclic\ alkene}$), 1432 ($\delta_{C\text{-}H\ alkane,\ methyl\ group}$), 1359 ($\delta_{O\text{-}H\ phenol}$), 1056 ($\nu_{C\text{-}N\ amine}$).

m.p.: 162.8-164.1°C.

3. NMR Characterisation

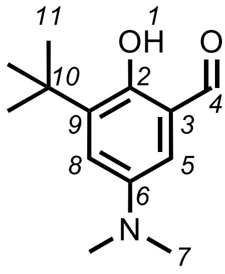

17

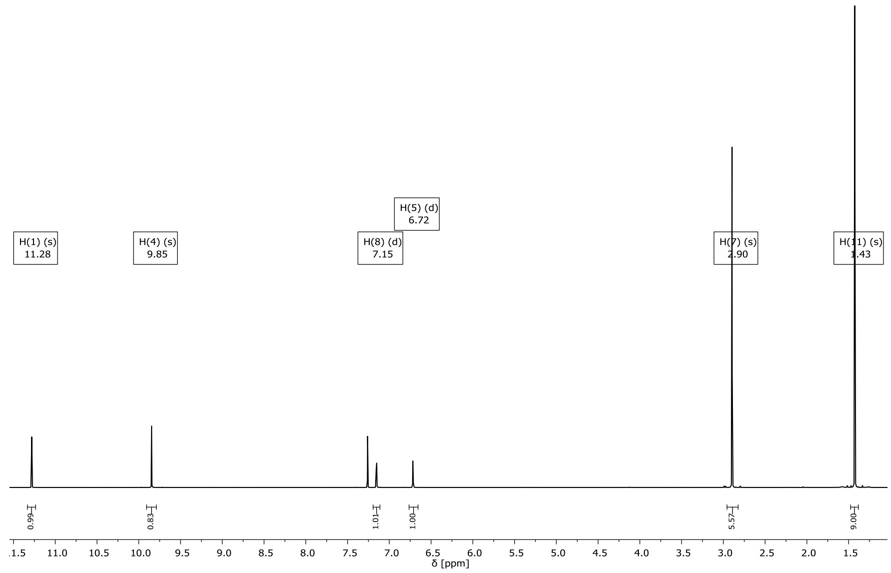

Figure S1 700 MHz ^{1}H-NMR of **17** in $CDCl_3$.

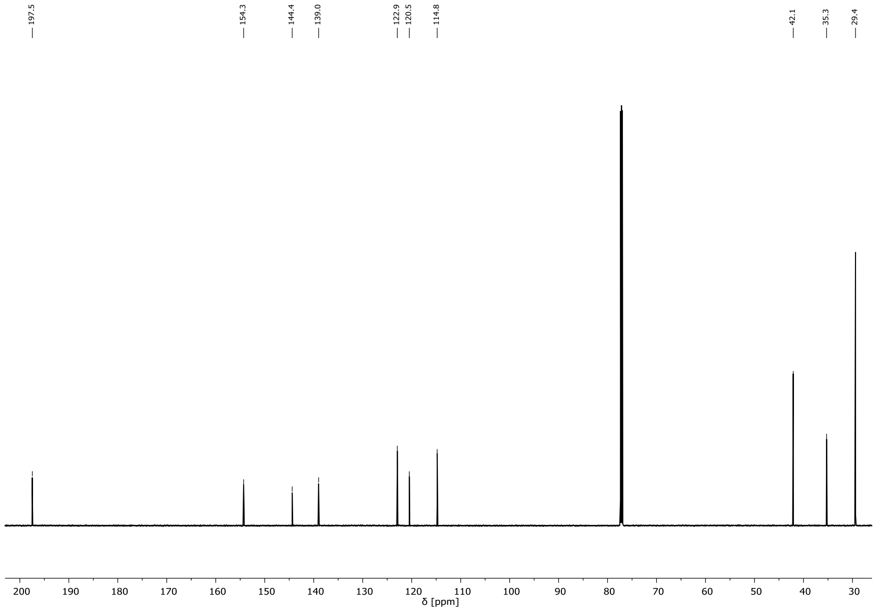

Figure S2 176 MHz ^{13}C-NMR of **17** in $CDCl_3$.

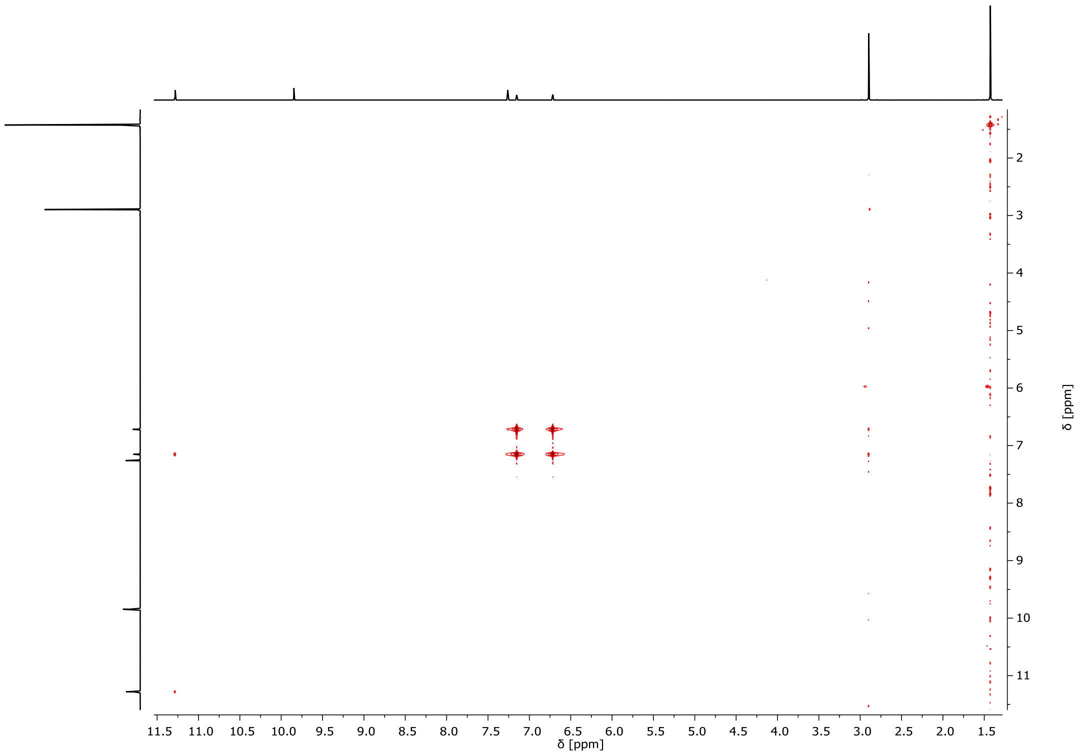

Figure S3 700 MHz ^1H-^1H COSY spectrum of **17** in $CDCl_3$.

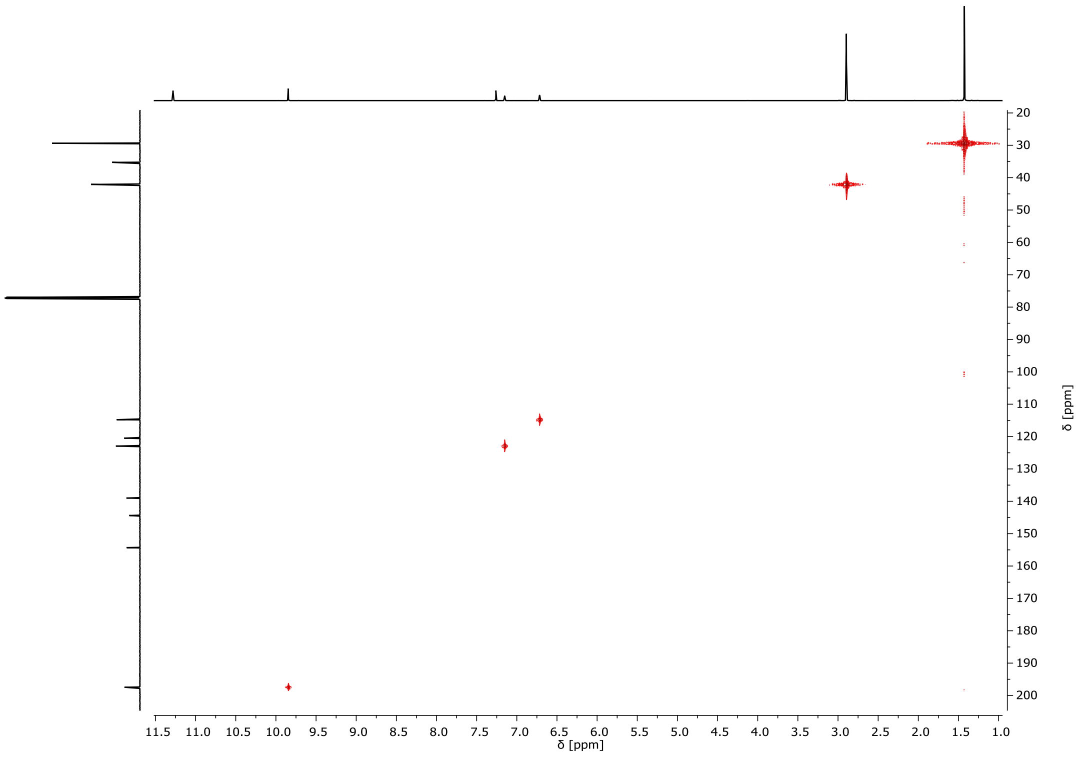

Figure S4 700 MHz ^1H-^{13}C Heteronuclear Single Quantum Coherence (HSQC) spectrum of **17** in $CDCl_3$.

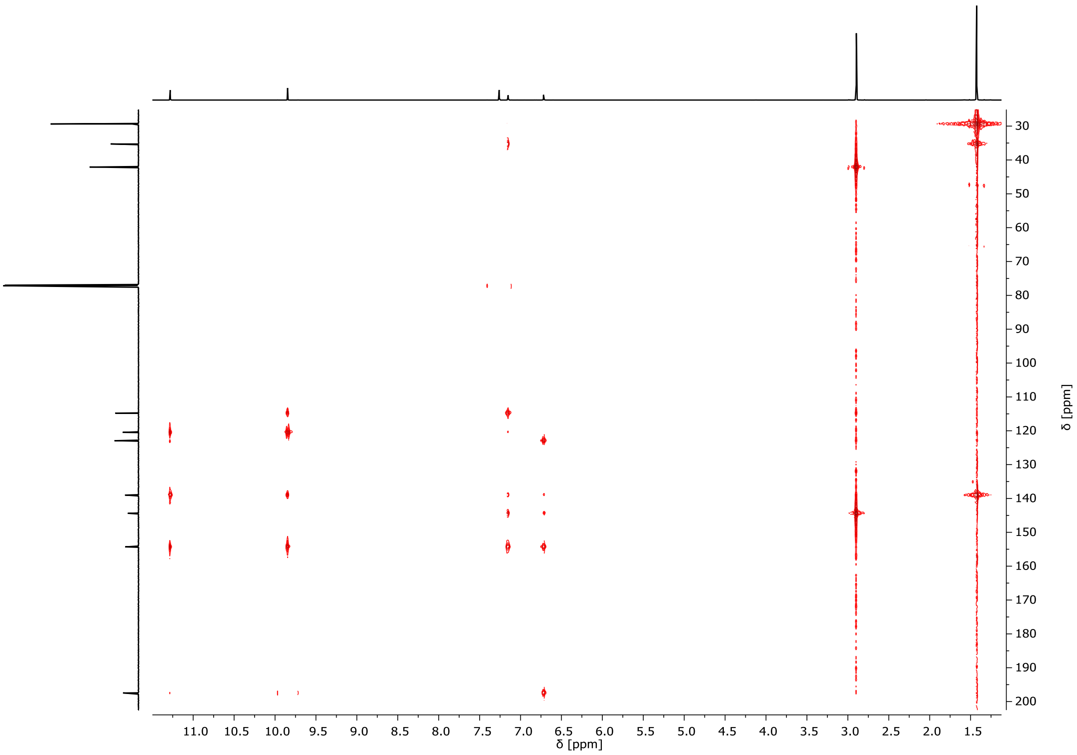

Figure S5 700 MHz ^{1}H-^{13}C Heteronuclear Multiple Bond Correlation (HMBC) spectrum of **17** in $CDCl_3$.

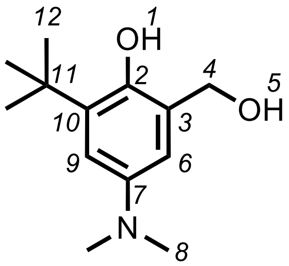

5

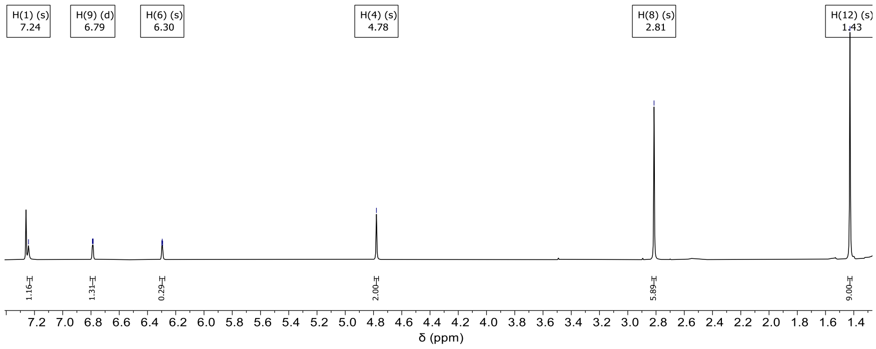

Figure S6 600 MHz ^{1}H-NMR of **5** in $CDCl_3$.

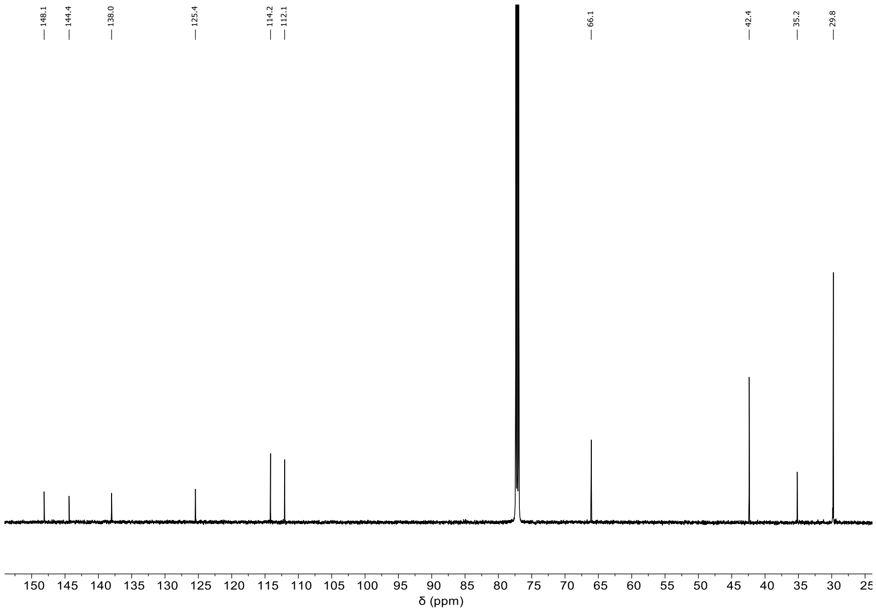

Figure S7 151 MHz ^{13}C-NMR of **5** in $CDCl_3$.

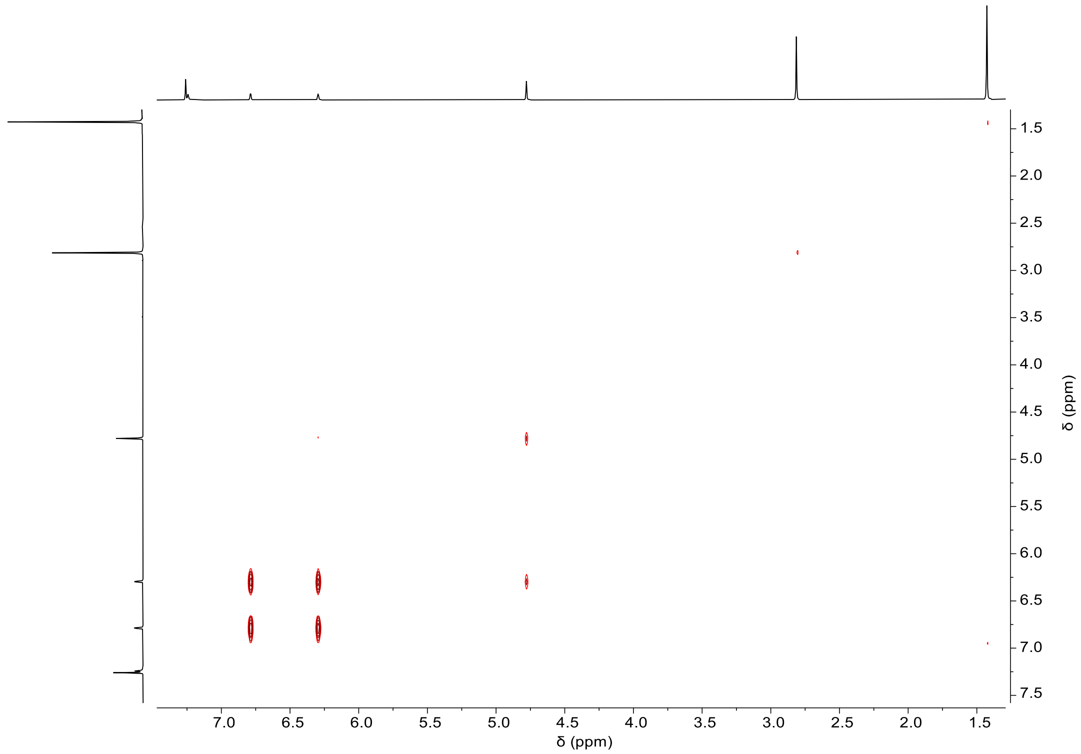

Figure S8 600 MHz ^{1}H-^{1}H COSY spectrum of **5** in $CDCl_3$.

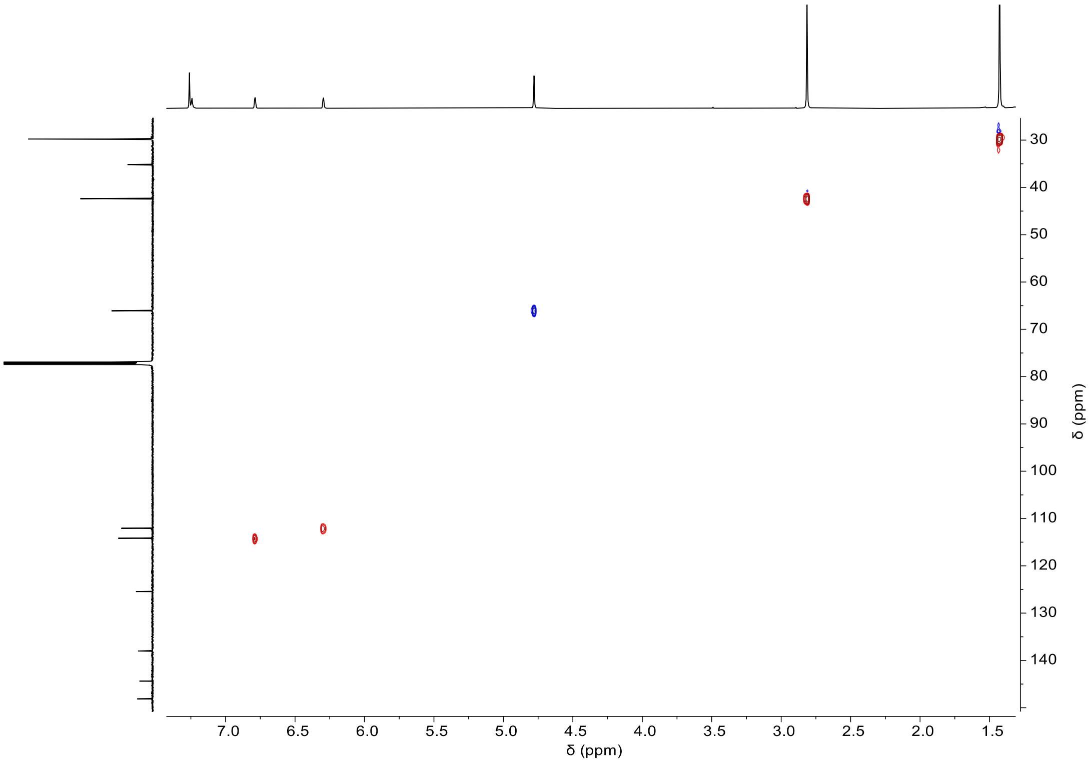

Figure S9 600 MHz ^{1}H-^{13}C Heteronuclear Single Quantum Coherence (HSQC) spectrum of **5** in $CDCl_3$.

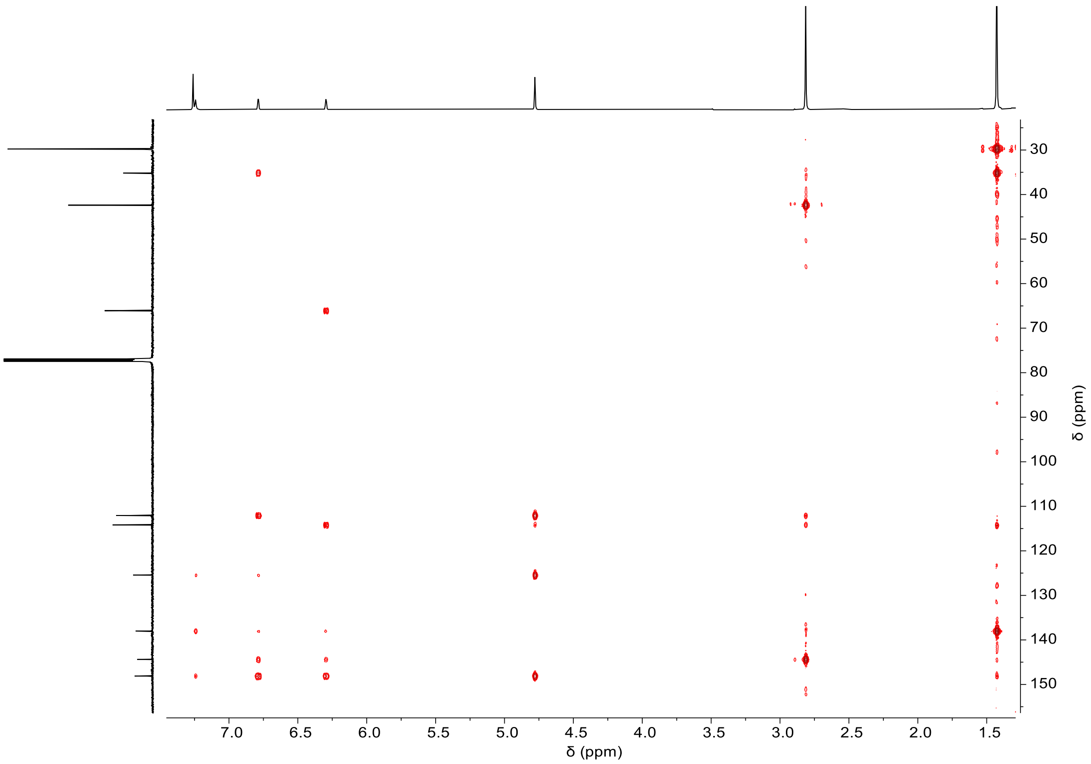

Figure S10 600 MHz ^{1}H-^{13}C Heteronuclear Multiple Bond Correlation (HMBC) spectrum of **5** in $CDCl_3$.

^{1}H-NMR of reference: benzyl alcohol

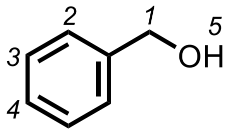

11

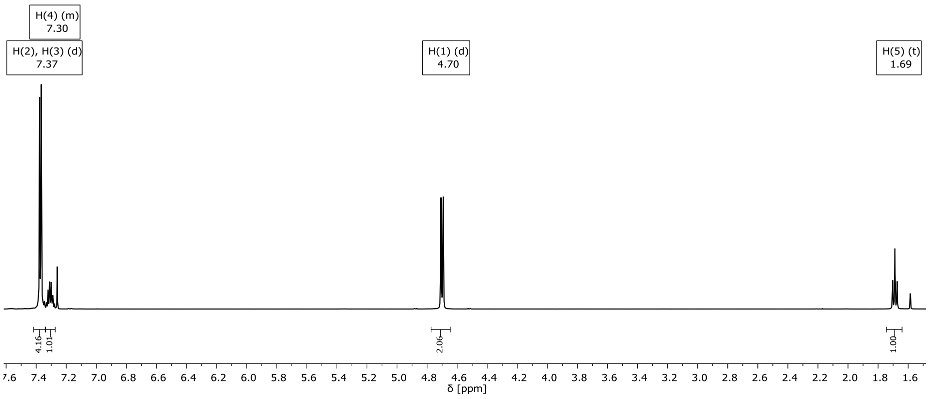

Figure S11 400 MHz ^{1}H-NMR of benzyl alcohol in $CDCl_3$.

4. Single-Crystal X-ray Crystallography

X-ray data were collected on a Bruker D8-QUEST diffractometer, equipped with an Incoatec IμS Cu microsource (λ = 1.5418 Å) and a PHOTON-III detector operating in shutterless mode. The crystal temperature was held at 180(2) K using an Oxford Cryosystems open-flow N_2 Cryostream. The control and processing software was Bruker *APEX4* (ver. 2021.4-0). Structures were solved using *SHELXT*[4] and refined using *SHELXL*.[5]

Structure 1 (displacement ellipsoids at 50% probability):

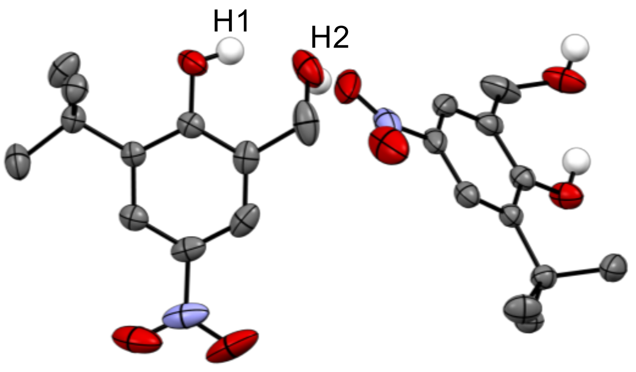

H atoms of both OH groups were located in the difference Fourier map and refined freely with isotropic displacement parameters.

**D**	**H**	**A**	**D–H (Å)**	**H···A (Å)**	**D···A (Å)**	**D–H···A (°)**	
O1	H1	O2	0.90(3)	1.85(3)	2.678(2)	152(3)	Intramolecular
O2	H2	$O3^{i}$	0.88(3)	2.30(3)	2.994(2)	136(3)	Intermolecular
O2	H2	$O4^{i}$	0.88(3)	2.53(3)	3.391(3)	165(3)	Intermolecular

Symmetry code (i): x – ½, –y + 1, –z + ½

Structure 3 (displacement ellipsoids at 50% probability):

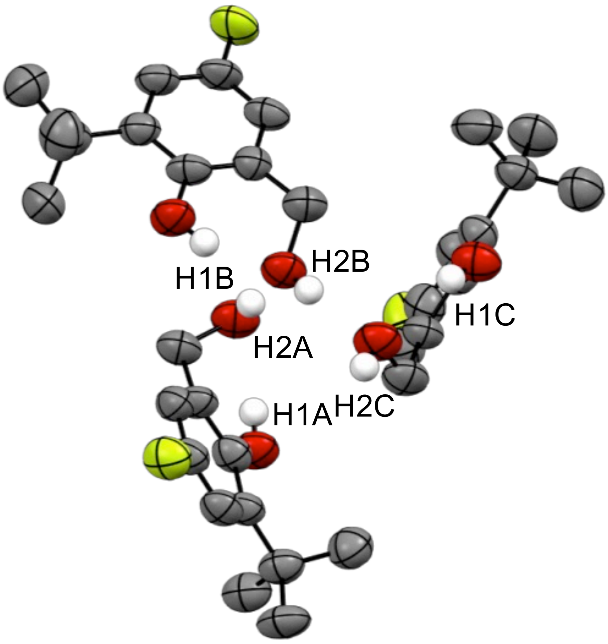

The crystal was a thin needle (min. dimension = 0.02 mm) and diffraction was weak: I/σ(I) falls below 3.0 around 1.25 Å. The data are integrated optimistically to 0.95 Å, where I/ σ(I) = 0.5, and the precision of the structure is limited accordingly. The structure contains three molecules in the asymmetric unit. H atoms of all OH groups are placed in idealised positions (they are not located from the X-ray data). The intramolecular H-bonds are unambiguous because there are no competing H-bond acceptors anywhere in the vicinity of O1 in each molecule. The intermolecular H-bonds form chains running along the *a* axis, and the chains could run in either direction.

**D**	**H**	**A**	**D–H (Å)**	**H···A (Å)**	**D···A (Å)**	**D–H···A (°)**	
O1A	H1A	O2A	0.84	2.04	2.776(6)	145.7	Intramolecular
O2A	H2A	O2B	0.85	1.84	2.687(7)	179.4	Intermolecular
O1B	H1B	O2B	0.84	1.91	2.638(6)	144.2	Intramolecular
O2B	H2B	O2C	0.84	1.88	2.685(7)	160.0	Intermolecular
O1C	H1C	O2C	0.84	2.01	2.724(6)	142.3	Intramolecular
O2C	H2C	O2A[ii]	0.84	1.94	2.696(6)	148.4	Intermolecular

Symmetry code (ii): x + 1, y, z

Table S1 Summary of the crystal and refinement details.

	**1**	**3**
CCDC Deposition Number	2419978	2419977
Cambridge data number	CH_B1_0045	CH_B1_0050
Chemical formula	$C_{11}H_{15}NO_4$	$C_{11}H_{15}FO_2$
Formula weight	225.24	198.23
Temperature / K	180(2)	180(2)
Crystal system	orthorhombic	triclinic
Space group	P c c n	P –1
a / Å	13.7547(6)	6.5600(11)
b / Å	19.2286(9)	14.795(3)
c / Å	8.4326(4)	17.736(3)
alpha / °	90	100.253(6)
beta / °	90	98.746(6)
gamma / °	90	100.247(6)
Unit-cell volume / $Å^3$	2230.28(18)	1636.6(5)
Z	8	6
Calc. density / g cm^{-3}	1.342	1.207
F(000)	960	636
Radiation type	Cu Ka	Cu Ka
Absorption coefficient / mm^{-1}	0.856	0.772
Crystal size / mm^3	0.14 x 0.06 x 0.02	0.30 x 0.02 x 0.02
2-Theta range / °	7.90-133.28	5.16-108.34
Completeness to max 2-theta	0.998	0.976
No. of reflections measured	17509	15674
No. of independent reflections	1964	3889
R(int)	0.0693	0.1999
No. parameters / restraints	157 / 0	389 / 0
Final R1 values (I > 2s(I))	0.0421	0.0986
Final wR(F2) values (all data)	0.1087	0.2314
Goodness-of-fit on F2	1.052	0.959
Largest difference peak & hole / e $Å^{-3}$	0.233, -0.243	0.203, -0.195

Geometry optimisation using dispersion-correct DFT calculations:

All crystal structures referred to in the text were energy minimised using dispersion-corrected density functional theory (DFT-D) calculations. These optimisation methods have been established to reproduce correct crystal structures.[6] The principal purpose in this case was to provide robust and comparable positions for the H atoms in the structures. For the lower-resolution crystal structure of **3**, the observation of minimal deviation on minimisation also adds further confidence to the validity of the structure. The geometrical parameters reported for the H-bonds in the text are taken from the minimised structures.

Prior to minimisation, the positions of H atoms in the crystal structures were normalised using the default settings in *Mercury*.[7] The calculations were then made using *CASTEP*[8] via the interface in *Materials Studio*.[9] The PBE exchange-correlation functional was applied,[10] with a dispersion correction according to Grimme.[11] The plane-wave basis-set cut-off was set to 340 eV and all other parameters were set to the "Fine" defaults in *Materials Studio*. Unit-cell parameters were constrained in each case to those from the reported crystal structure and the space-group symmetry was imposed.

The following table shows the maximum and rms average Cartesian deviation for all atoms and all non-H atoms on minimisation. The RMS Cartesian displacement for all atoms lies in the range 0.030-0.112 Å, well within the estimated upper limit of 0.25 Å for correct crystal structures.[6] Structures **3**, MeBr and MeMe show larger displacements for individual H atoms; these correspond to rotational displacements of Me groups.

	RMS non-H Cartesian displacement (Å)	Max non-H Cartesian displacement (Å)	RMS Cartesian displacement (Å)	Max Cartesian displacement (Å)	Max displacement atom (Å)
**1**	0.083	0.133	0.092	0.142	H9C
**3**	0.066	0.123	0.100	0.279	H10E
**4**	0.025	0.065	0.049	0.138	H8
MeBr	0.082	0.132	0.105	0.281	H11D
MeF	0.016	0.031	0.030	0.092	H2A
MeMe	0.080	0.128	0.112	0.310	H11B
MeNO2	0.053	0.156	0.060	0.156	O3

5. NMR Experiments

^{1}H-NMR Titration – General Procedure

A diluted solution of the host (5 mL) in *n*-octane is prepared from the stock solution of the receptor in *n*-octane. 600 µL of the host solution is titrated with a solution of the guest (G) containing also the host at the same concentration in *n*-octane. A ^{1}H-NMR spectrum with WET[12] or presat[13] solvent suppression is recorded for every point of the titration on a 500 MHz spectrometer. The chemical shifts of the protons of the host are monitored upon addition of various concentrations of guest. The observed chemical shift (δ_{obs}, ppm, Equation 1) is a weighted average of the chemical shifts of the free host (δ_H, ppm) and the host-guest complex ($\delta_{H\cdot G}$, ppm):

$$\delta_{obs} = \delta_H X_H + \delta_{H\cdot G} X_{H\cdot G} \quad (1)$$

where X_H and $X_{H\cdot G}$ are the mole fractions of H ($X_H = [H]/[H]_0$) and of H·G ($X_{H\cdot G} = [H\cdot G]/[H]_0$), respectively. $[H]_0$ is the initial concentration of H ($[H]_0 = [H\cdot G] + [H]$) and $[G]_0$ is the initial concentration of G ($[G]_0 = [H\cdot G] + [G]$). Since $X_H = 1 - X_{H\cdot G}$, equation (1) can be rearranged:

$$\delta_{obs} = \delta_H (1 - X_{H\cdot G}) + \delta_{H\cdot G} X_{H\cdot G} \quad (2)$$

$$\frac{\delta_{obs} - \delta_H}{\delta_{HG} - \delta_H} = X_{H\cdot G} = \frac{[HG]}{[H]_0} \quad (3)$$

Given that the association constant K_a for the 1:1 H·G complex is $[H\cdot G]/[H][G]$ and $[H]_0 = [H\cdot G] + [H]$, equation (4) can be written as

$$\frac{\delta_{obs} - \delta_H}{\delta_{HG} - \delta_H} = \frac{K_a[G]}{1 + K_a[G]} \quad (4)$$

and [G] can be determined using equation (5) by making iteratively guesses of K_a and solving for [G] until the theoretical isotherm matches the experimental data:

$$K_a[G]^2 + (K_a[H]_0 - K_a[G]_0 + 1)[G] - [G]_0 = 0 \quad (5)$$

A Microsoft Excel spreadsheet with purpose-written VBA macros was used to solve equations (4) and (5).[14]

Each titration was repeated three times, fitted with equations (4) and (5), and an average value of the association constant along with its standard error (with 95% confidence) is reported in Table S2.

Donor	Acceptor<br>Quin
**5**	$(4.2 \pm 0.1)x10^2$
benzyl alcohol	17 ± 4 M^{-1}

Table S2 Association constants (M^{-1}) for formation of 1:1 complexes measured by NMR spectroscopy titrations in *n*-octane at 298 K. Errors are the standard error of the mean of three independent experiments.

^{1}H-NMR Dilution – General Procedure

A diluted solution of the host (2 mL) in *n*-octane is prepared from the stock solution of the receptor in *n*-octane. Increasing volumes of the host solution were added to 600 μL of *n*-octane. Alternatively, to 600 μL of a diluted solution of the host, increasing volumes of *n*-octane were added. A ^{1}H-NMR spectrum with WET[12] or presat[13] solvent suppression is recorded after each addition.

Molecule 1

Dilution Experiment of 1 in *n*-octane

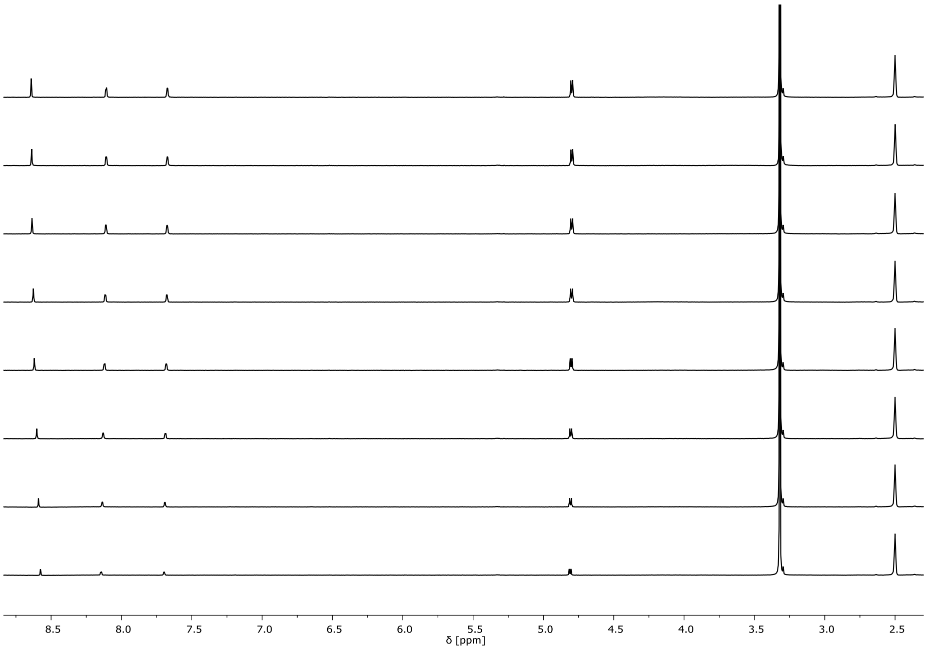

Figure S12 NMR dilution of **1** in *n*-octane (500 MHz ^{1}H-NMR spectra with WET solvent suppression, at the following concentrations of **1** (from bottom to top) 0.153 mM, 0.211 mM, 0.260 mM, 0.339 mM, 0.377 mM, 0.421 mM, 0.435 mM, 0.446 mM).

Titration of 1 with quinuclidine in *n*-octane

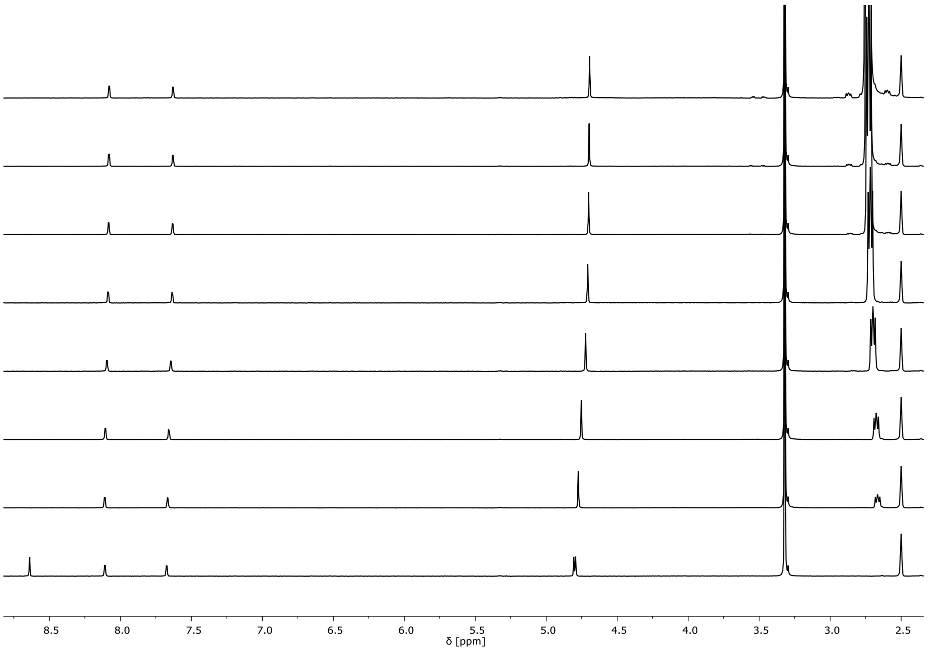

Figure S13 500 MHz ^{1}H-NMR titration of **1** (0.446 mM) with quinuclidine in *n*-octane (500 MHz ^{1}H-NMR spectra with WET solvent suppression, H_2O at 3.32 ppm; DMSO at 2.50 ppm).

Molecule 2

Dilution Experiment of 2 in *n*-octane

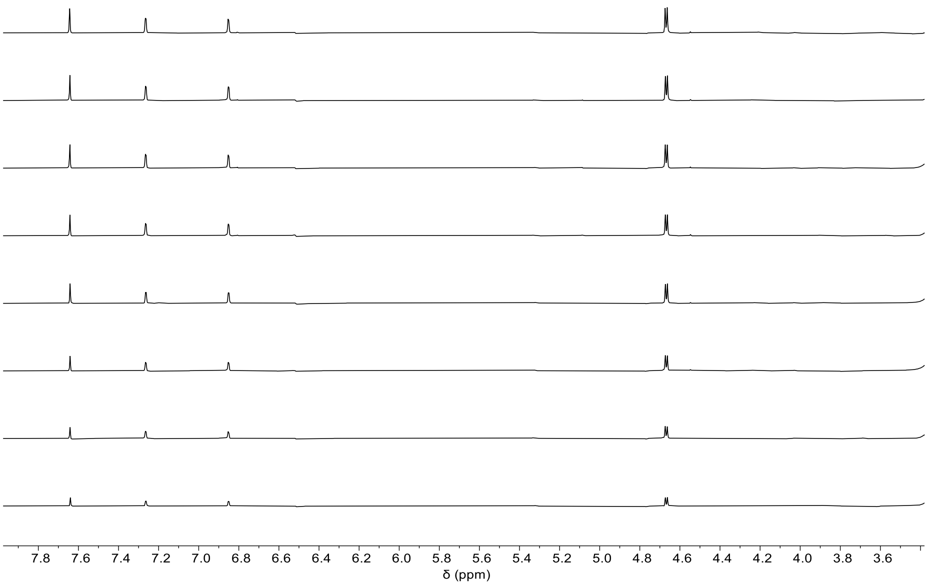

Figure S14 NMR dilution of **2** in *n*-octane (600 MHz ^{1}H-NMR spectra with WET solvent suppression, at the following concentrations of **2** (from bottom to top) 0.069 mM, 0.094 mM, 0.117 mM, 0.152 mM, 0.169 mM, 0.189 mM, 0.195 mM, 0.200 mM).

Titration of 2 with quinuclidine in *n*-octane

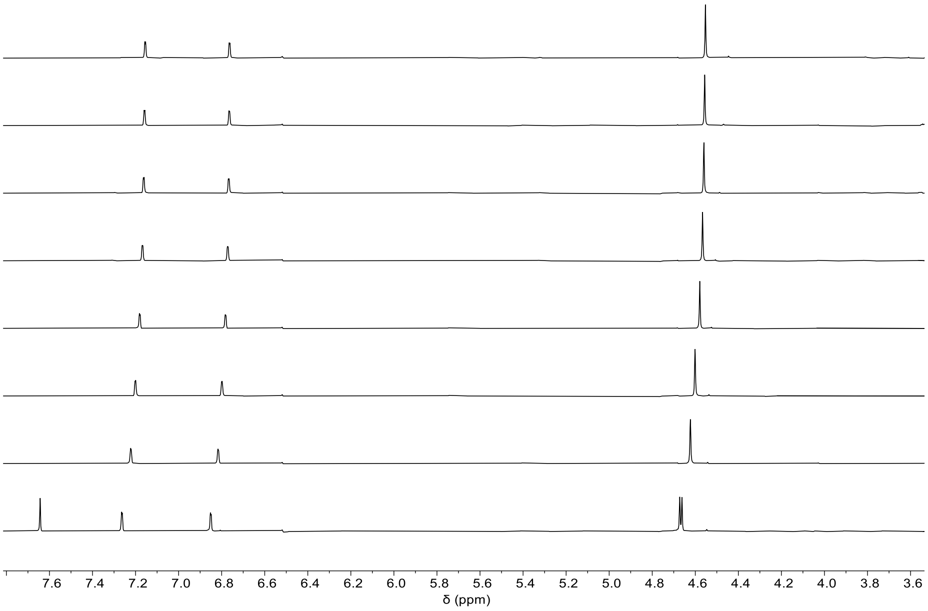

Figure S15 600 MHz ^{1}H-NMR titration of **2** (0.200 mM) with quinuclidine in *n*-octane (600 MHz ^{1}H-NMR spectra with WET solvent suppression).

Molecule 3

Dilution Experiment of 3 in *n*-octane

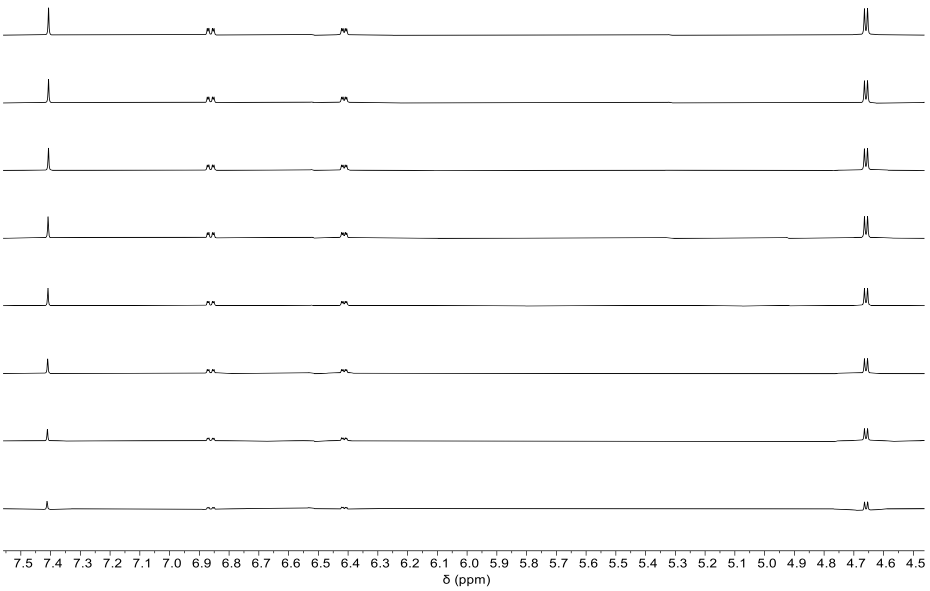

Figure S16 NMR dilution of **3** in *n*-octane (600 MHz ^{1}H-NMR spectra with WET solvent suppression, at the following concentrations of **3** (from bottom to top) 0.069 mM, 0.094 mM, 0.117 mM, 0.152 mM, 0.169 mM, 0.189 mM, 0.195 mM, 0.200 mM).

Titration of 3 with quinuclidine in *n*-octane

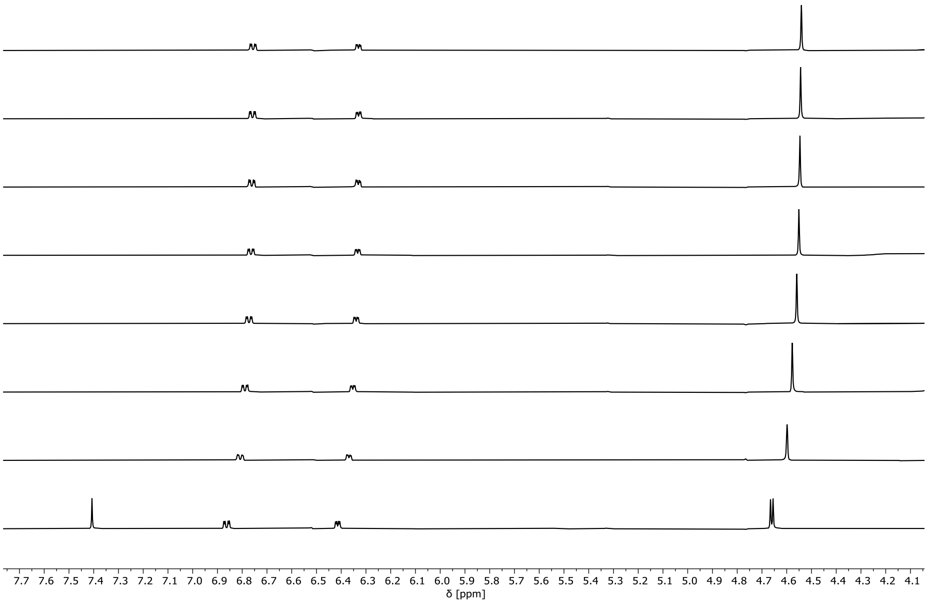

Figure S17 600 MHz ^{1}H-NMR titration of **3** (0.200 mM) with quinuclidine in *n*-octane (600 MHz ^{1}H-NMR spectra with WET solvent suppression).

Molecule 4

Dilution Experiment of 4 in *n*-octane

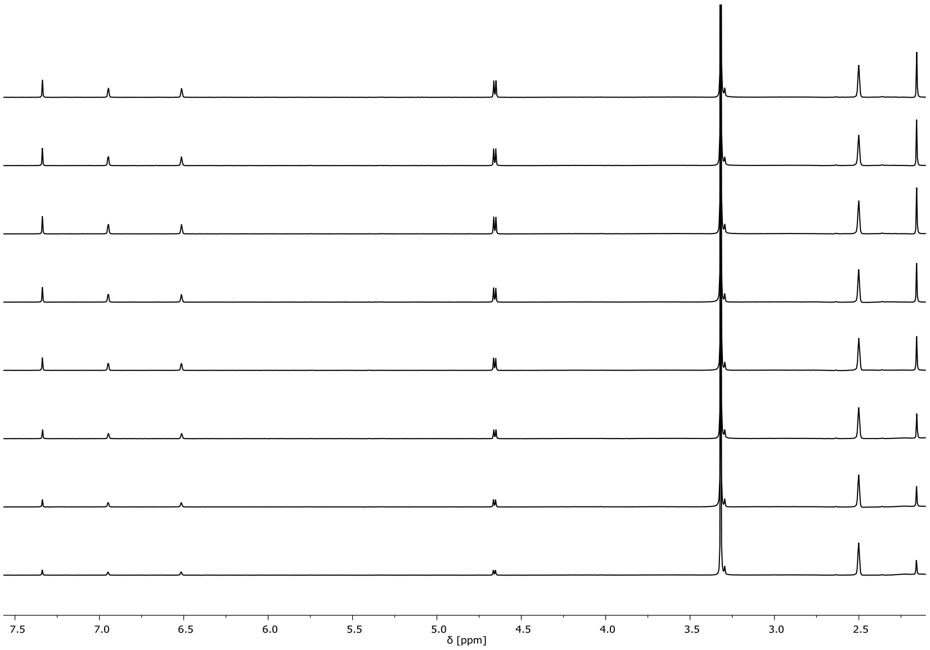

Figure S18 NMR dilution of **4** in *n*-octane (500 MHz ^{1}H-NMR spectra with WET solvent suppression, at the following concentrations of **4** (from bottom to top) 0.161 mM, 0.223 mM, 0.274 mM, 0.358 mM, 0.398 mM, 0.445 mM, 0.460 mM, 0.471 mM).

Titration of 4 with quinuclidine in *n*-octane

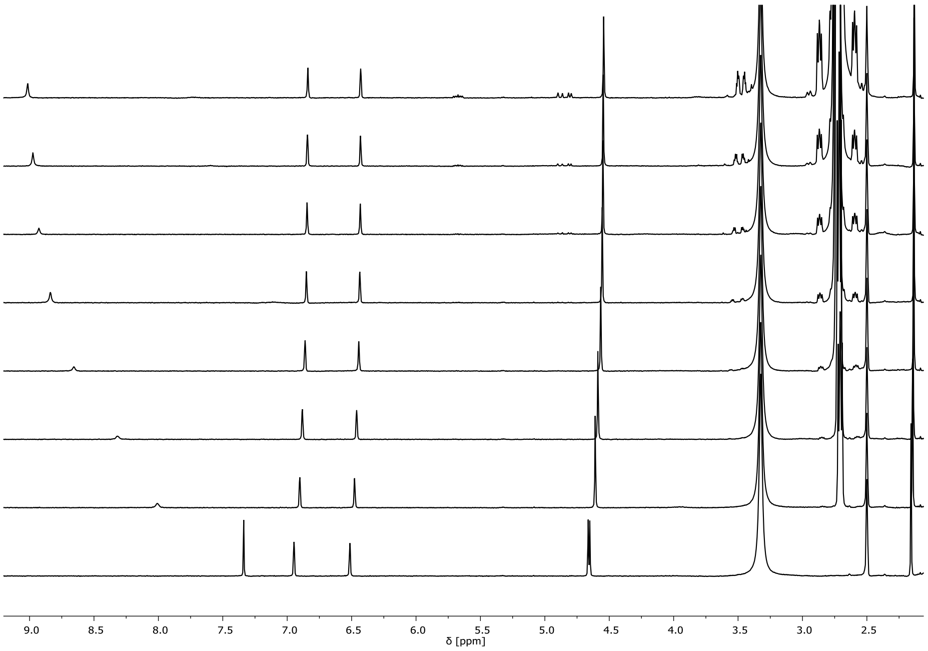

Figure S19 500 MHz ^{1}H-NMR titration of **4** (0.471 mM) with quinuclidine in *n*-octane (500 MHz ^{1}H-NMR spectra with WET solvent suppression, H_2O at 3.32 ppm; DMSO at 2.50 ppm).

Molecule 5

Dilution Experiment of 5 in *n*-octane

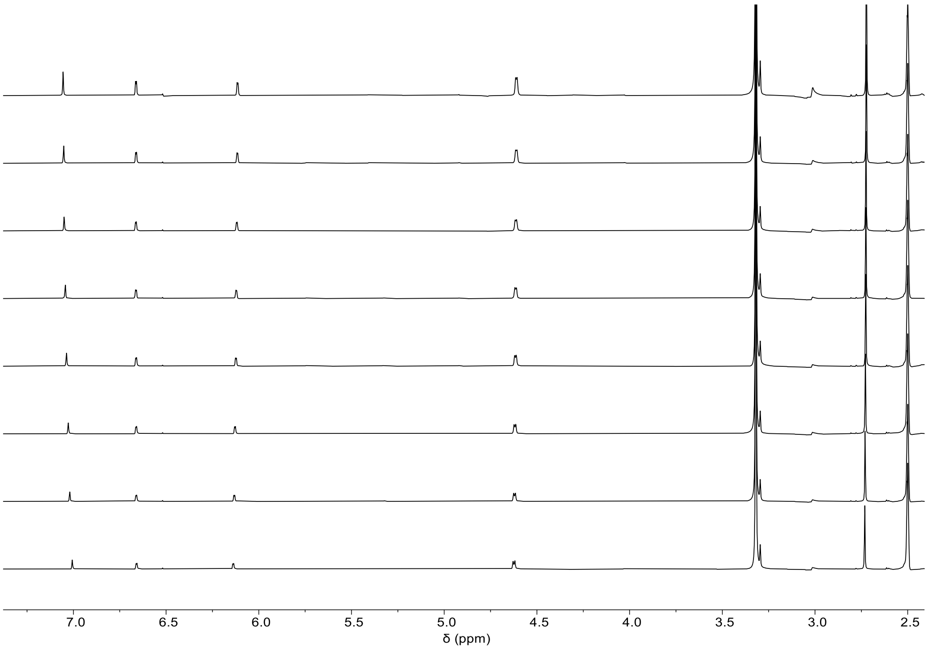

Figure S20 NMR dilution of **5** in *n*-octane (600 MHz ^{1}H-NMR spectra with WET solvent suppression, at the following concentrations of **5** (from bottom to top) 0.117 mM, 0.138 mM, 0.152 mM, 0.169 mM, 0.179 mM, 0.190 mM, 0.197 mM, 0.250 mM).

Titration of 5 with quinuclidine in *n*-octane

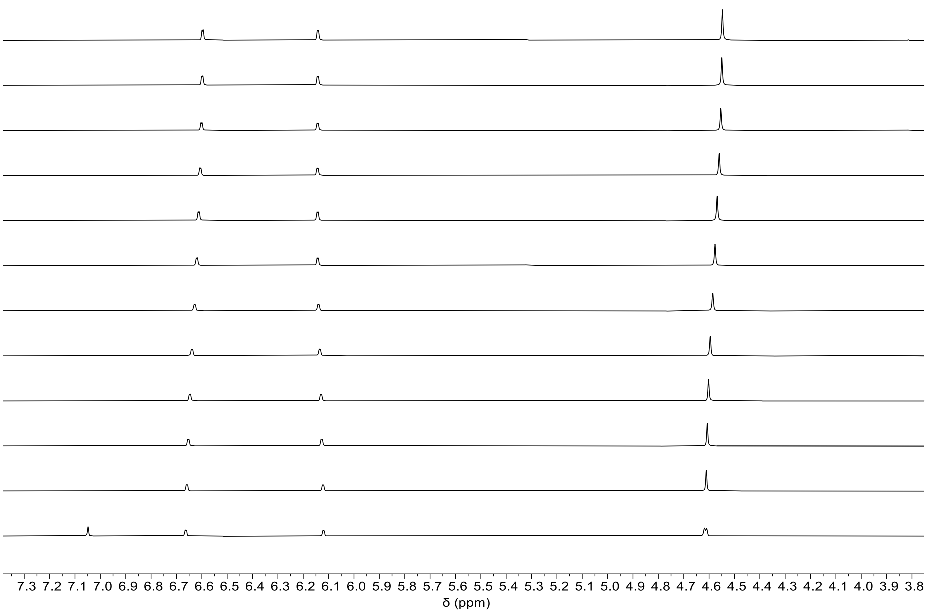

Figure S21 600 MHz ^{1}H-NMR titration of **5** (0.250 mM) with quinuclidine in *n*-octane (600 MHz ^{1}H-NMR spectra with WET solvent suppression).

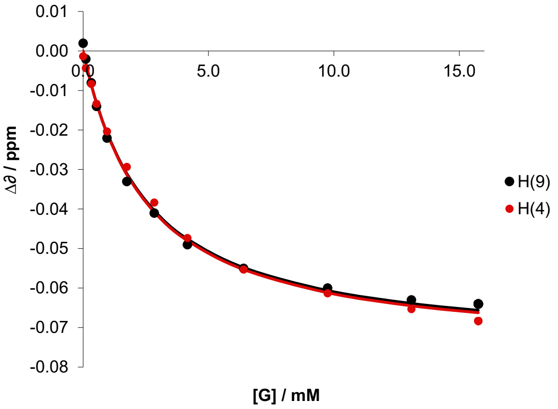

Figure S22 Fitting of the data from the NMR titration of **5** with quinuclidine in *n*-octane (Figure S21) with a 1:1 binding model.

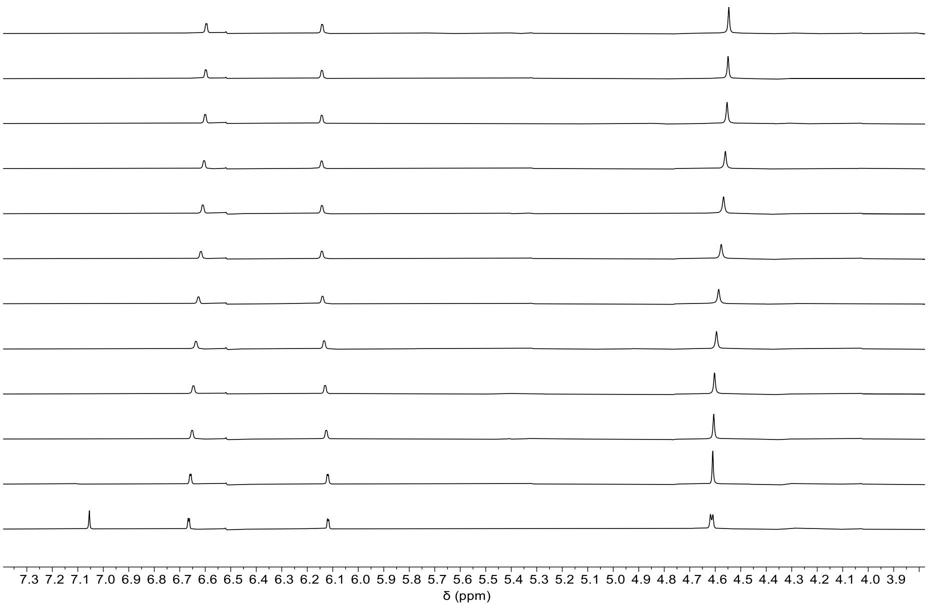

Figure S23 600 MHz ^{1}H-NMR titration of **5** (0.250 mM) with quinuclidine in *n*-octane (600 MHz ^{1}H-NMR spectra with WET solvent suppression).

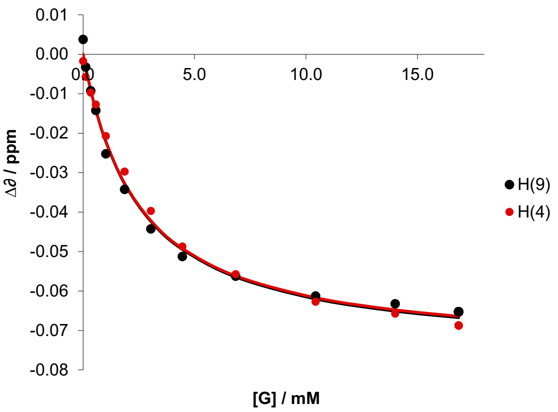

Figure S24 Fitting of the data from the NMR titration of **5** with quinuclidine in *n*-octane (Figure S23) with a 1:1 binding model.

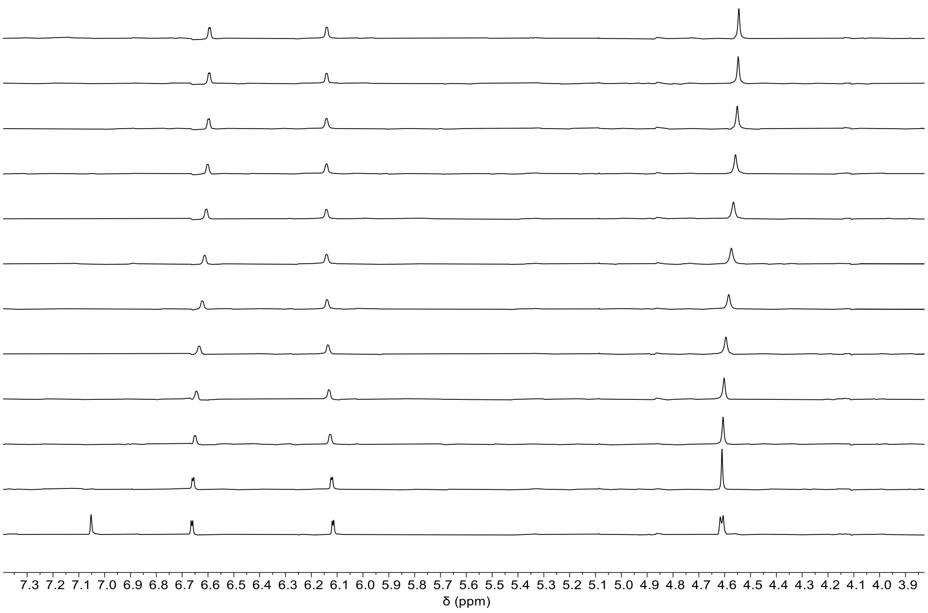

Figure S25 500 MHz ^{1}H-NMR titration of **5** (0.250 mM) with quinuclidine in *n*-octane (500 MHz ^{1}H-NMR spectra with WET solvent suppression).

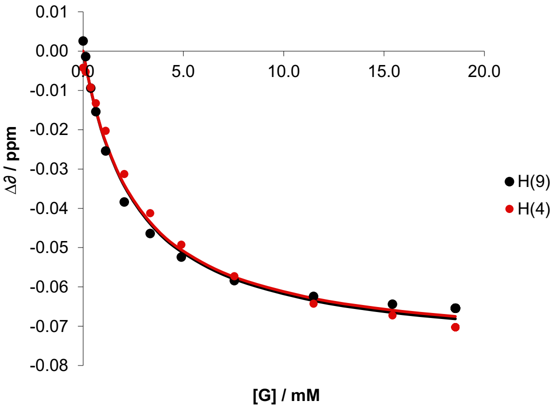

Figure S26 Fitting of the data from the NMR titration of **5** with quinuclidine in *n*-octane (Figure S25) with a 1:1 binding model.

Benzyl alcohol

Dilution Experiment of benzyl alcohol in *n*-octane

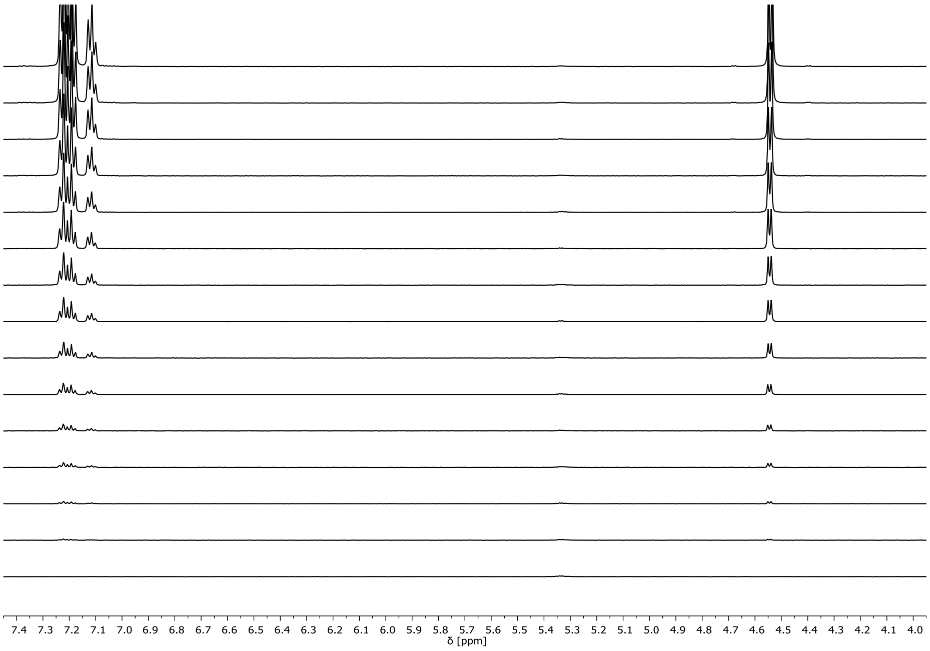

Figure S27 NMR dilution of benzyl alcohol in *n*-octane (500 MHz ^{1}H-NMR spectra with presat solvent suppression, at the following concentrations of **11** (from bottom to top) 0 mM, 0.044 mM, 0.087 mM, 0.173 mM, 0.257 mM, 0.421 mM, 0.580 mM, 0.884 mM, 1.169 mM, 1.693 mM, 2.160 mM, 2.962 mM, 4.177 mM, 5.411 mM, 6.841 mM).

Titrations of benzyl alcohol with quinuclidine in *n*-octane

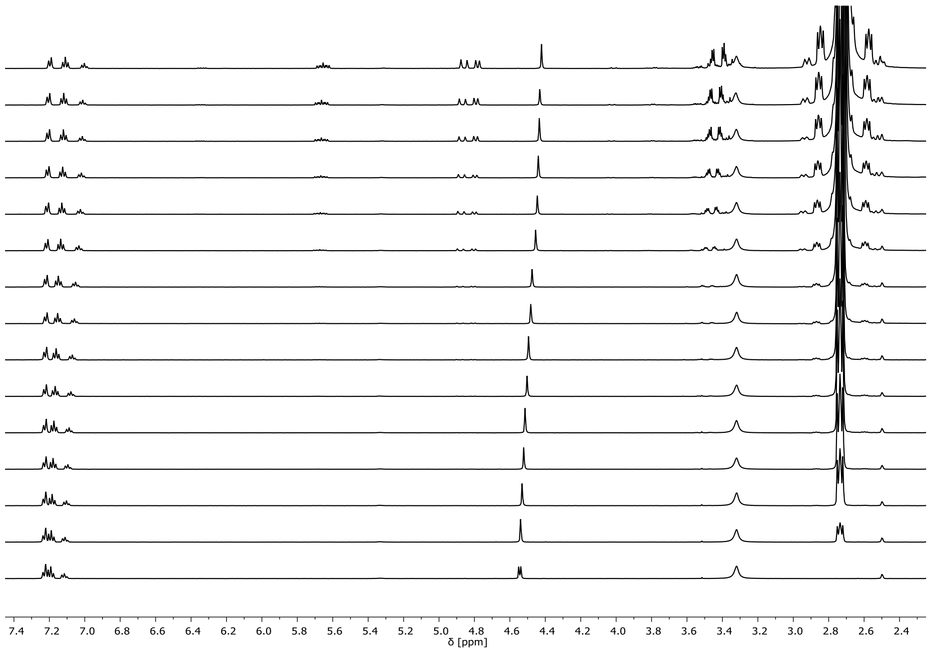

Figure S28 500 MHz ^{1}H-NMR titration of benzyl alcohol (1.000 mM) with quinuclidine in *n*-octane (500 MHz ^{1}H-NMR spectra with presat solvent suppression, H_2O at 3.32 ppm; DMSO at 2.50 ppm).

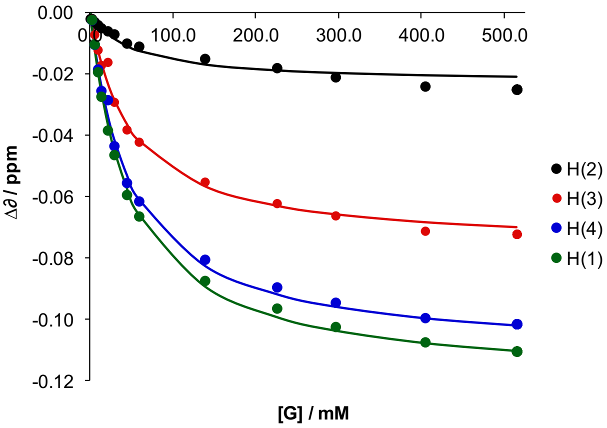

Figure S29 Fitting of the data from the NMR titration of benzyl alcohol with quinuclidine in *n*-octane (Figure S28) with a 1:1 binding model.

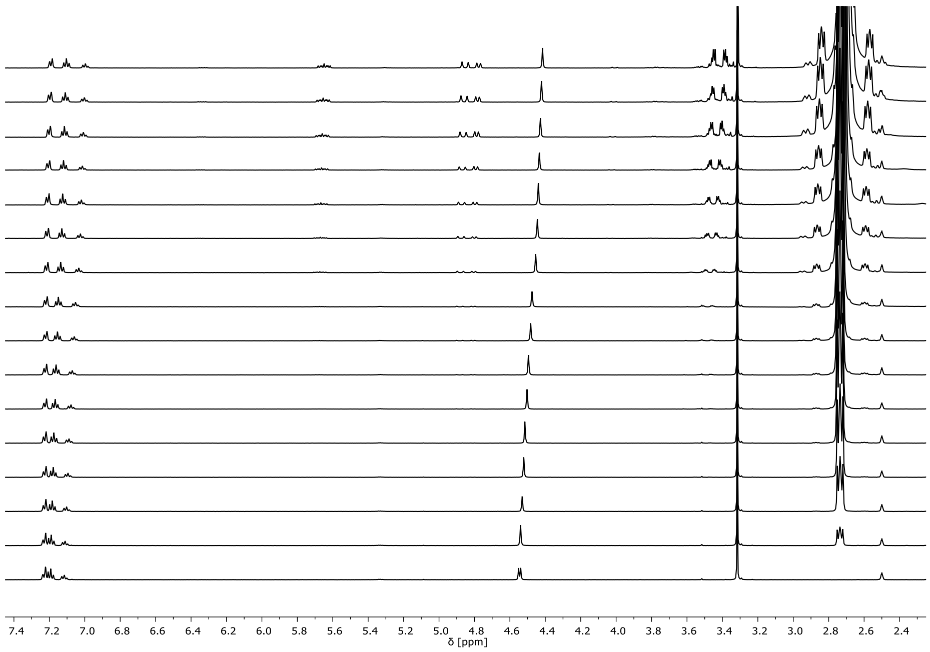

Figure S30 500 MHz ^{1}H-NMR titration of benzyl alcohol (1.000 mM) with quinuclidine in *n*-octane (500 MHz ^{1}H-NMR spectra with presat solvent suppression, H_2O at 3.32 ppm; DMSO at 2.50 ppm).

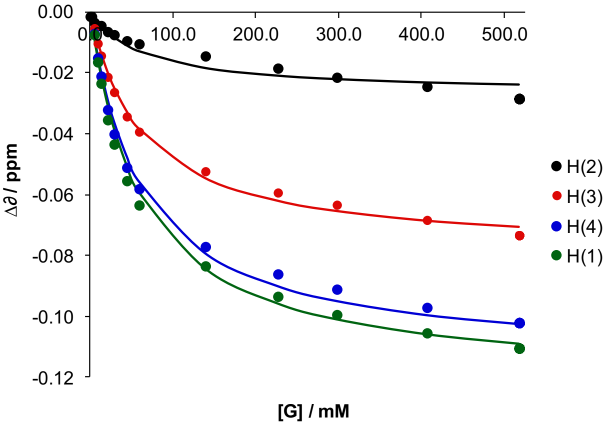

Figure S31 Fitting of the data from the NMR titration of benzyl alcohol with quinuclidine in *n*-octane (Figure S30) with a 1:1 fitting model.

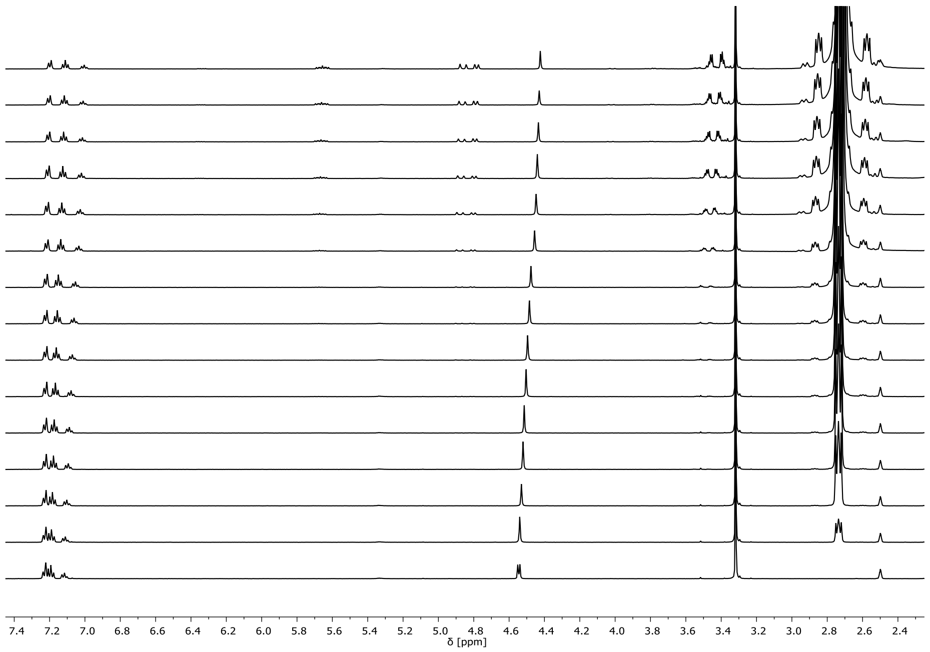

Figure S32 500 MHz ^{1}H-NMR titration of benzyl alcohol (1.000 mM) with quinuclidine in *n*-octane (500 MHz ^{1}H-NMR spectra with presat solvent suppression, H_2O at 3.32 ppm; DMSO at 2.50 ppm).

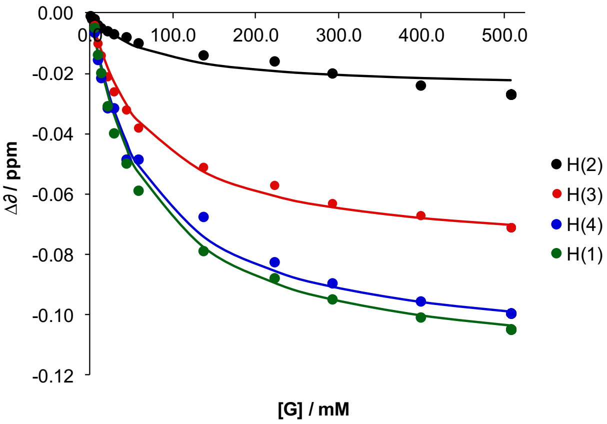

Figure S33 Fitting of the data from the NMR titration of benzyl alcohol with quinuclidine in *n*-octane (Figure S32) with a 1:1 binding model.

NOESY Experiments[15,16]

(a)

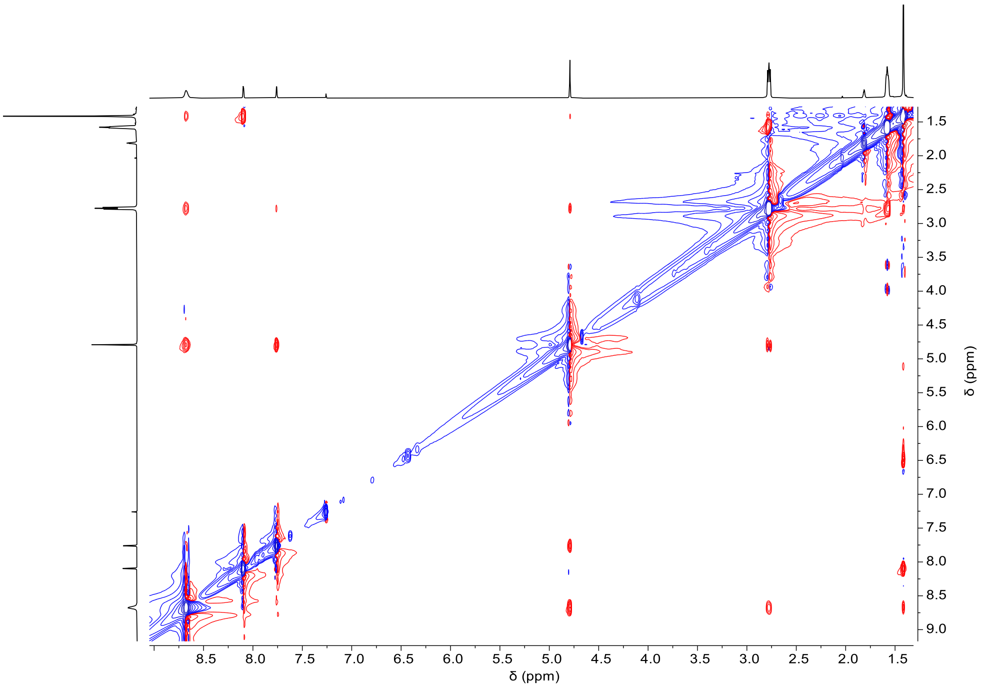

(b)

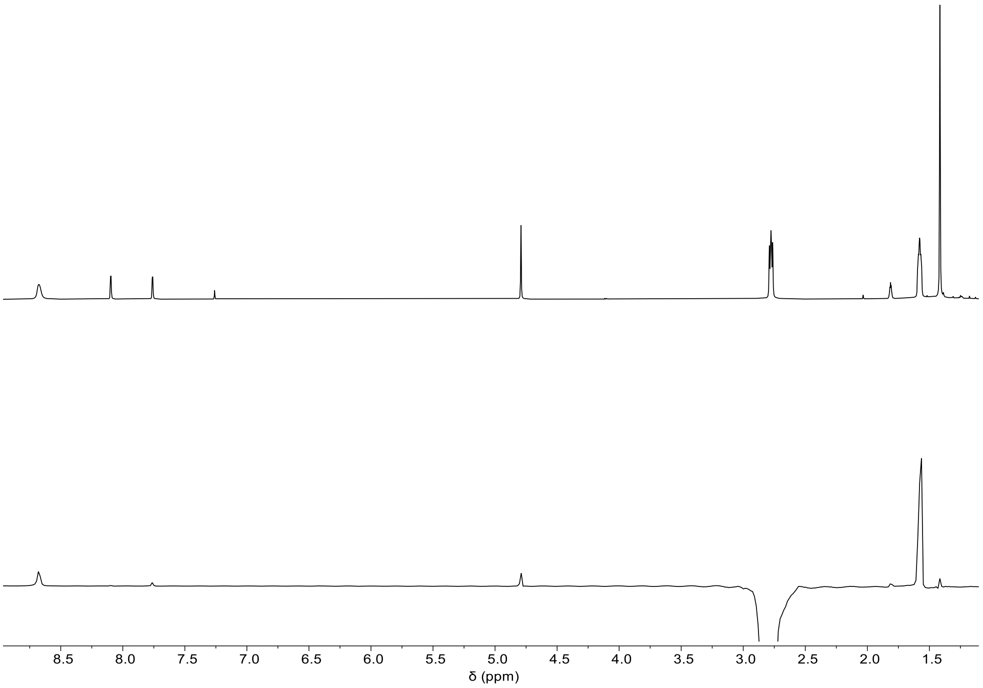

Figure S34 (a) 600 MHz NOESY experiment of **1** and quinuclidine (1:1) in $CDCl_3$, and (b) ^{1}H-NMR spectrum of **1** and quinuclidine (1:1) in $CDCl_3$ (on the top) and ^{1}H-NMR spectrum extracted from the NOESY experiment at 2.78 ppm.

(a)

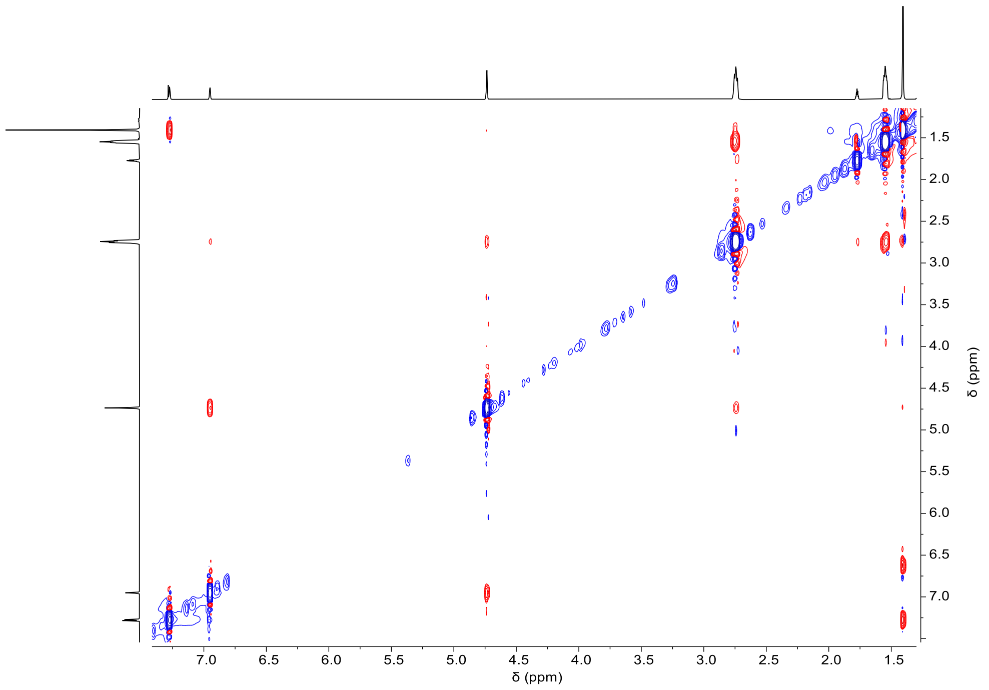

(b)

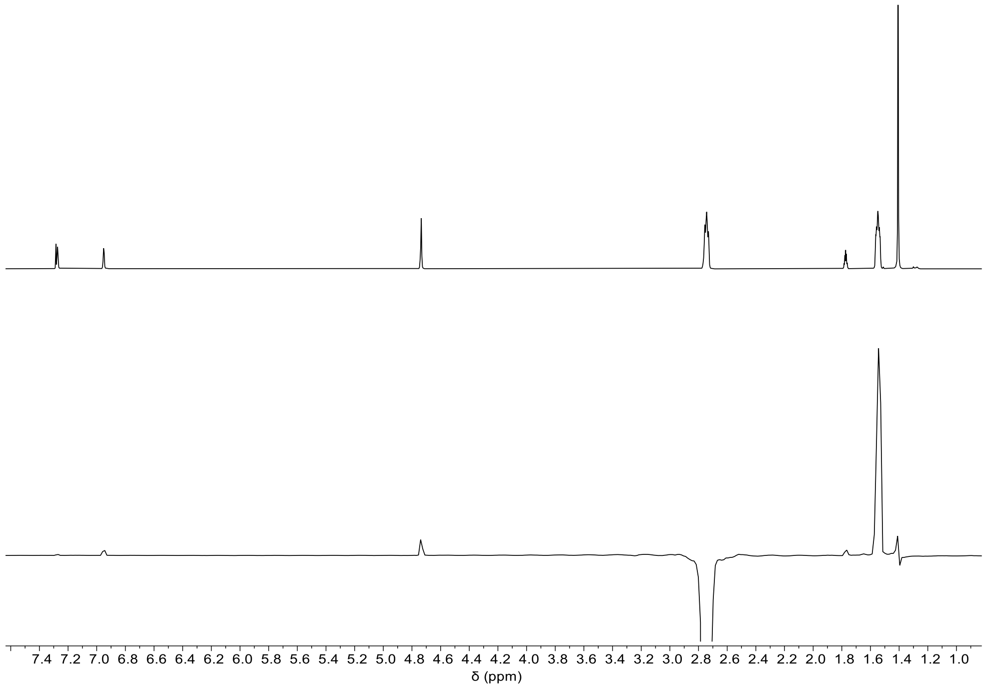

Figure S35 (a) 600 MHz NOESY experiment of **2** and quinuclidine (1:1) in $CDCl_3$, and (b) ^{1}H-NMR spectrum of **2** and quinuclidine (1:1) in $CDCl_3$ (on the top) and ^{1}H-NMR spectrum extracted from the NOESY experiment at 2.74 ppm.

(a)

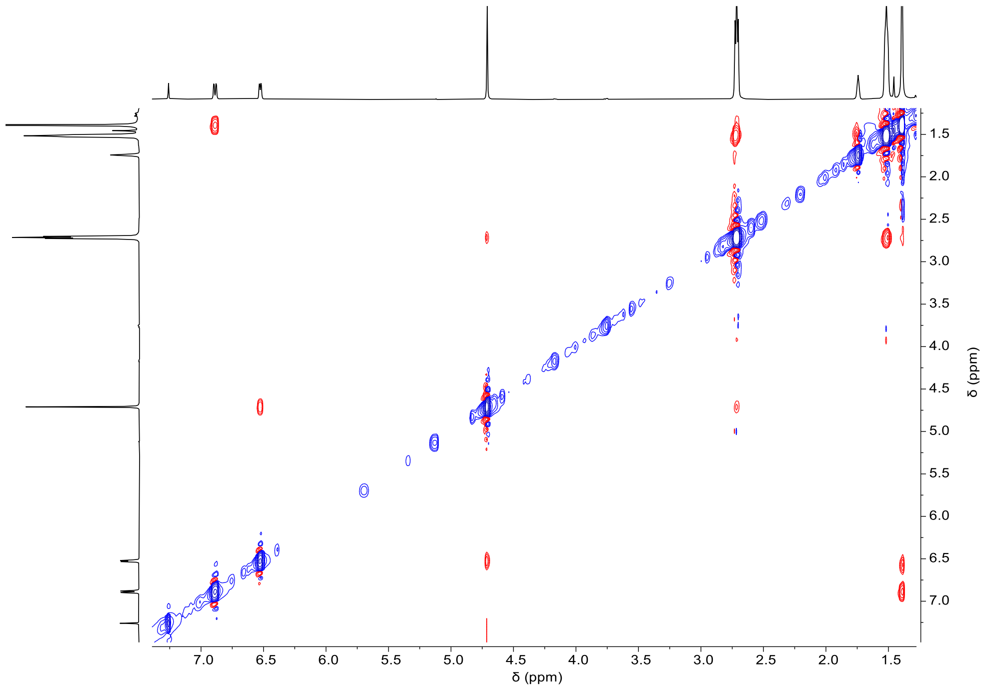

(b)

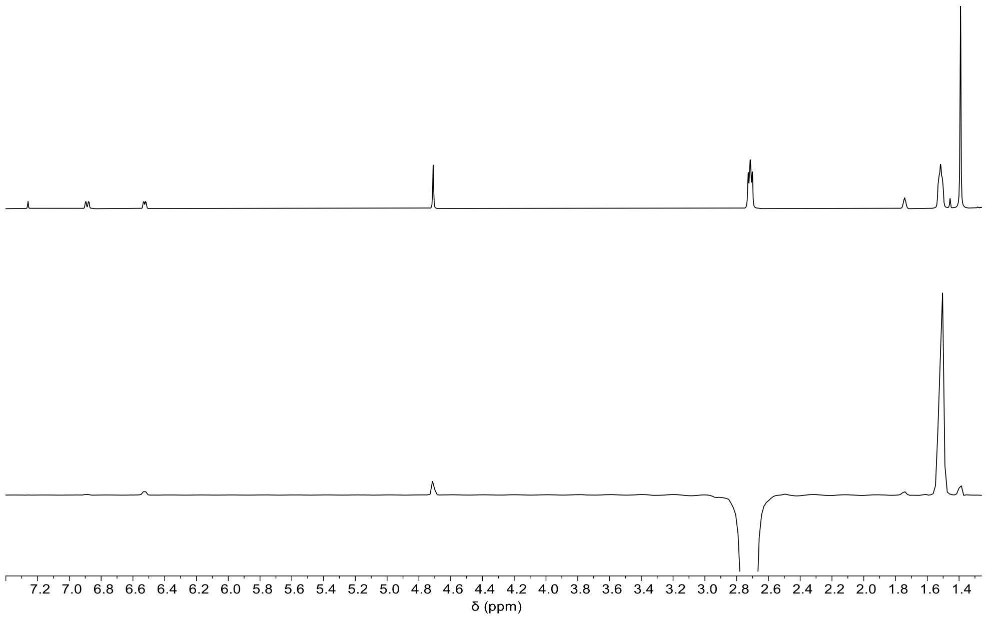

Figure S36 (a) 600 MHz NOESY experiment of **3** and quinuclidine (1:1) in $CDCl_3$, and (b) 700 MHz ^{1}H-NMR spectrum of **3** and quinuclidine (1:1) in $CDCl_3$ (on the top) and ^{1}H-NMR spectrum extracted from the NOESY experiment at 2.72 ppm.

(a)

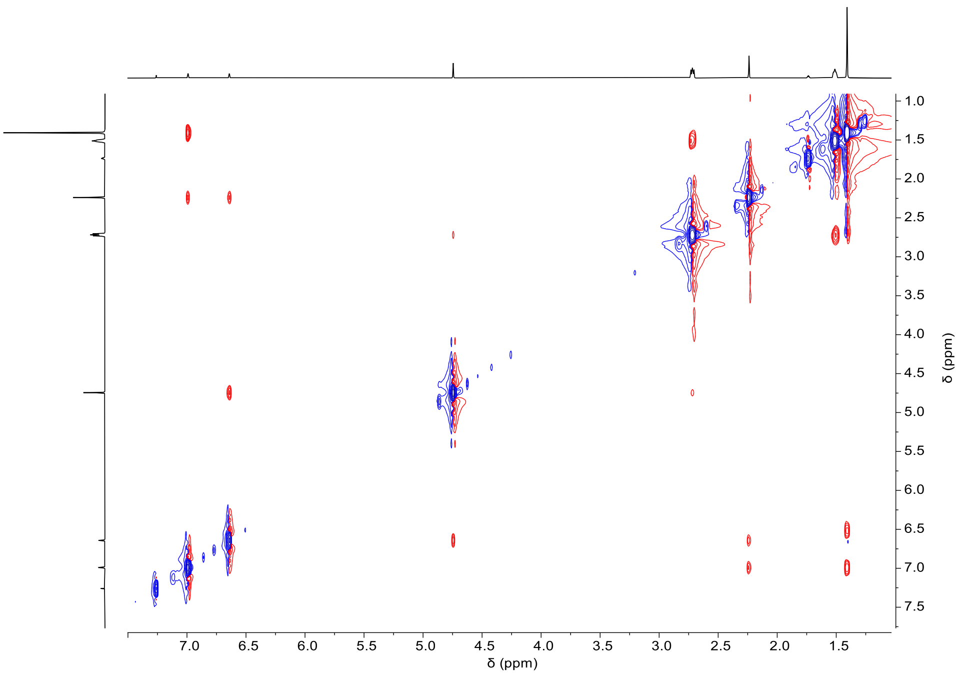

(b)

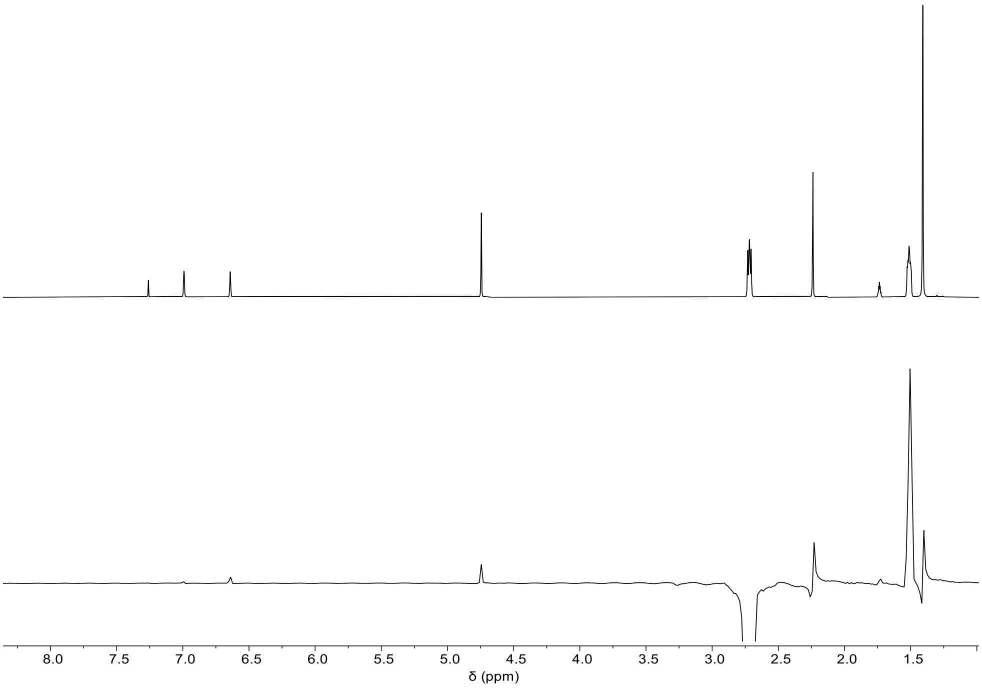

Figure S37 (a) 600 MHz NOESY experiment of **4** and quinuclidine (1:1) in $CDCl_3$, and (b) ^{1}H-NMR spectrum of **4** and quinuclidine (1:1) in $CDCl_3$ (on the top) and ^{1}H-NMR spectrum extracted from the NOESY experiment at 2.72 ppm.

(a)

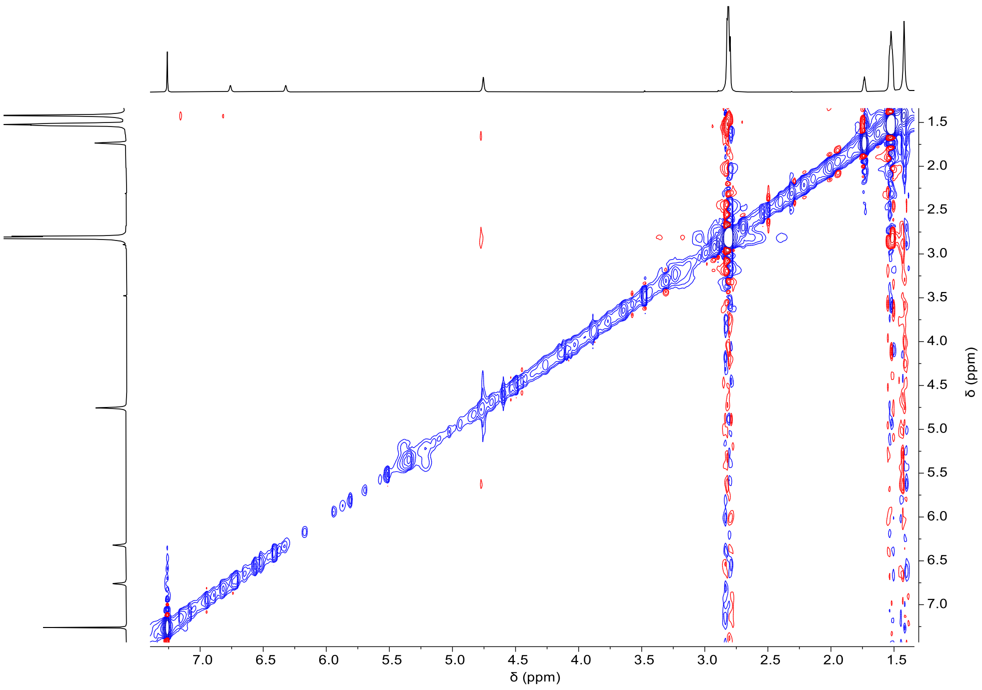

(b)

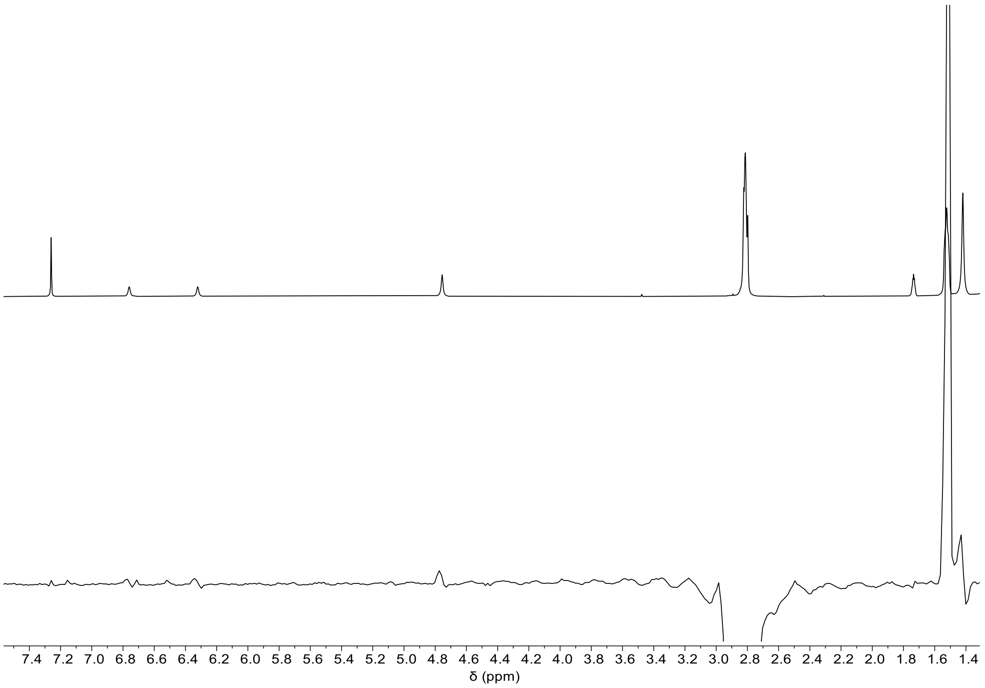

Figure S38 (a) 600 MHz NOESY experiment of **5** and quinuclidine (1:1) in $CDCl_3$, and (b) ^{1}H-NMR spectrum of **5** and quinuclidine (1:1) in $CDCl_3$ (on the top) and ^{1}H-NMR spectrum extracted from the NOESY experiment at 2.87 ppm.

6. UV-Vis Experiments

UV-Vis Titration – General Procedure

A diluted solution of the host (5 mL) in *n*-octane is prepared from the stock solution of the receptor in *n*-octane. 2 mL of the host solution is titrated with a solution of the guest containing also the host at the same concentration in *n*-octane. A UV-Vis spectrum is recorded for every point of the titration. Analogous to the NMR titrations, equation (6) can be written as

$$\frac{A_{obs}-A_0}{A_f-A_0} = \frac{K_a[G]}{1+K_a[G]} \tag{6}$$

where A_{obs} (a.u.) is the observed absorbance, A_0 (a.u.) is the initial absorbance, A_f (a.u.) is the final absorbance, K_a is the association constant and [G] is the concentration of free guest. [G] can be determined using equation (7) by making iteratively guesses of K_a and solving for [G] until the theoretical isotherm matches the experimental data:

$$K_a[G]^2+ (K_a[H]_0- K_a[G]_0+ 1)[G]- [G]_0=0 \tag{7}$$

where $[H]_0$ and $[G]_0$ are the total concentrations of the host and guest, respectively.
A Microsoft Excel spreadsheet with purpose-written VBA macros was used to solve equations (6) and (7) fitting the experimentally measured absorbance at specified wavelengths.

Each titration was repeated three times, fitted with equations (6) and (7), and an average value of the association constant along with its standard error (with 95% confidence) is reported in Table S3.

UV-Vis Dilution – General Procedure

A diluted solution of the host (5 mL) in *n*-octane is prepared from the stock solution of the receptor in *n*-octane. Increasing volumes of *n*-octane were added to 2 mL or 1.5 mL of the host solution. A UV-Vis spectrum is recorded after each addition.

Donor	Acceptor<br>Quin
**1**	$(4.4 \pm 0.3)x10^3$
**2**	$(9.3 \pm 0.9)x10^2$
**3**	$(8.3 \pm 0.4)x10^2$
**4**	$(4.7 \pm 0.7)x10^2$

Table S3 Association constants (M^{-1}) for formation of 1:1 complexes measured by UV-Vis spectroscopy titrations in *n*-octane at 298 K. Errors are the standard error of the mean of three independent experiments.

Molecule 1

Dilution Experiment of 1 in *n*-octane

(a) (b)

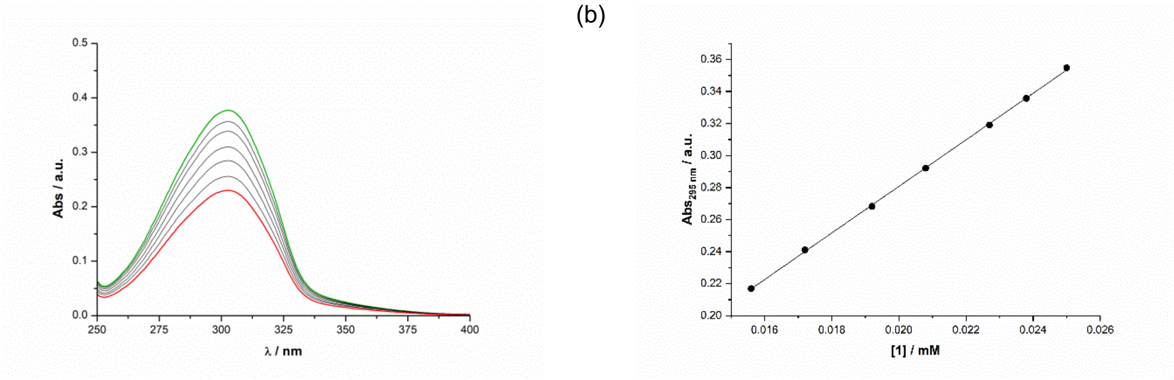

Figure S39 (a) UV-Vis spectra of **1** in *n*-octane at decreasing concentrations (from 0.025 mM in green to 0.016 mM in red), and (b) plot of the absorbance of **1** at 295 nm versus the concentration of **1** and its linear fitting.

Titrations of 1 with quinuclidine in *n*-octane

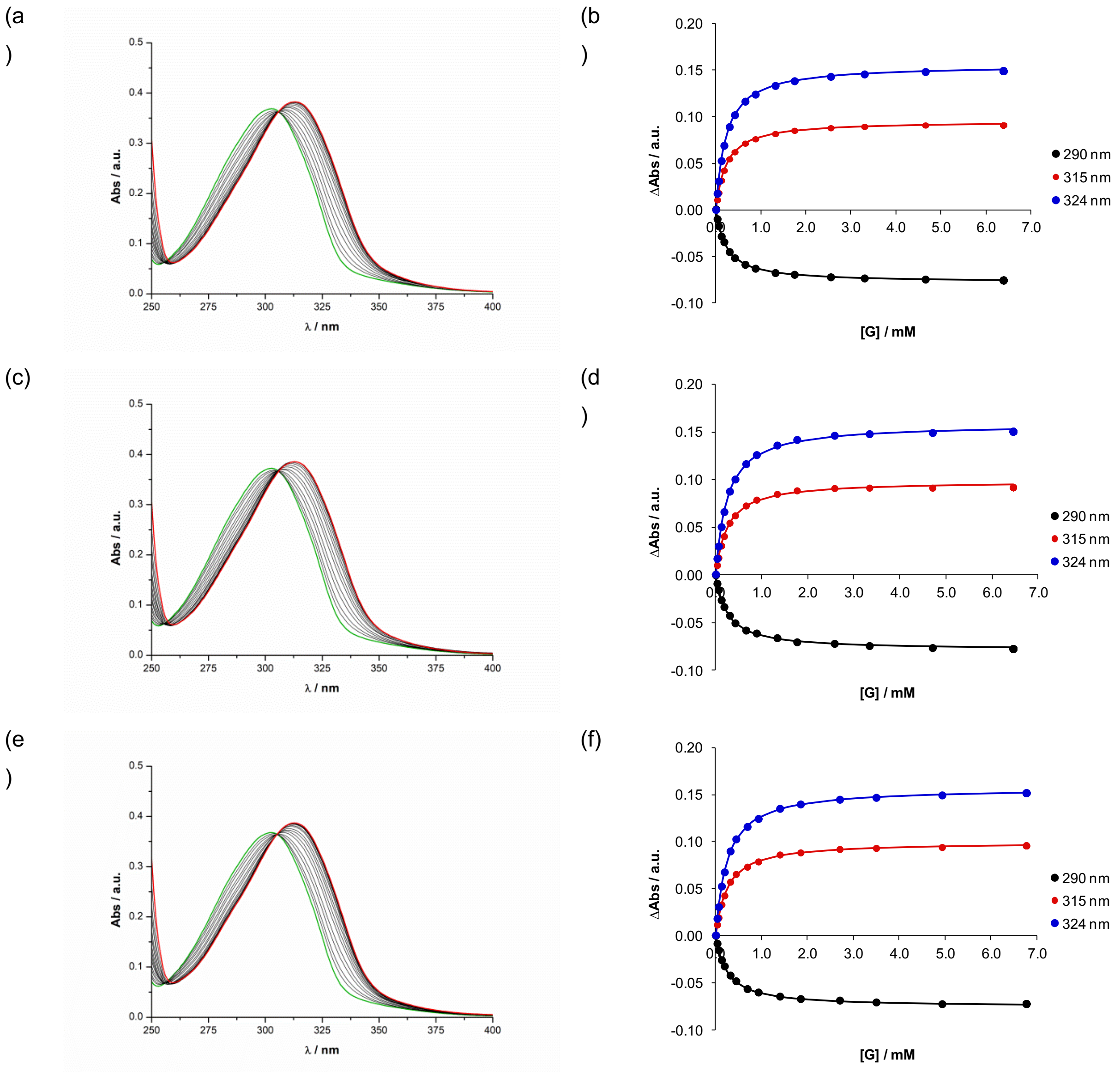

Figure S40 (a, c, e) UV-Vis spectra of **1** (0.025 mM in green) in *n*-octane at increasing concentrations of quinuclidine (from green to red), and (b, d, f) plot of the absorbance of **1** at 290 nm, 315 nm, 324 nm versus the concentration of **1** and its fittings to a 1:1 binding model.

Molecule 2

Dilution Experiment of 2 in *n*-octane

(a)

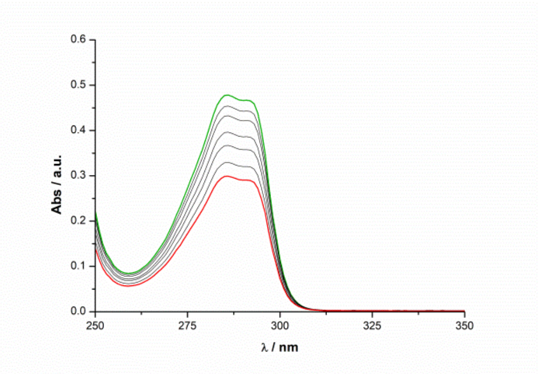

(b)

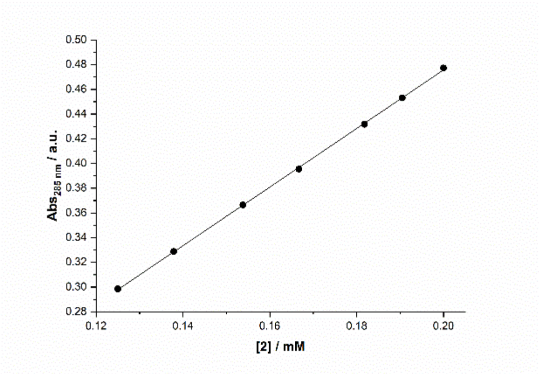

Figure S41 (a) UV-Vis spectra of **2** in *n*-octane at decreasing concentrations (from 0.200 mM in green to 0.125 mM in red), and (b) plot of the absorbance of **2** at 285 nm versus the concentration of **2** and its linear fitting.

Titrations of 2 with quinuclidine in *n*-octane

(a)

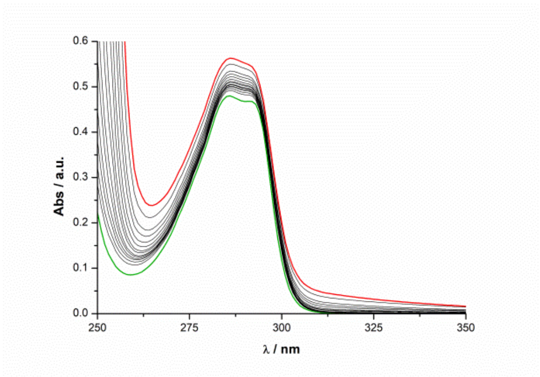

(b)

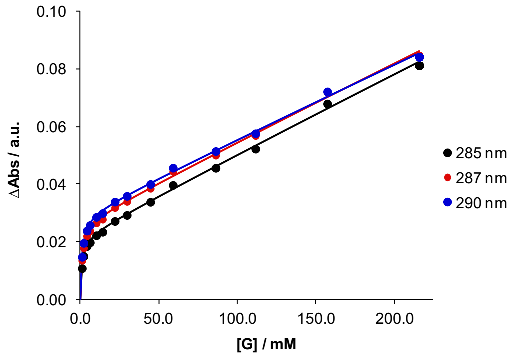

(c)

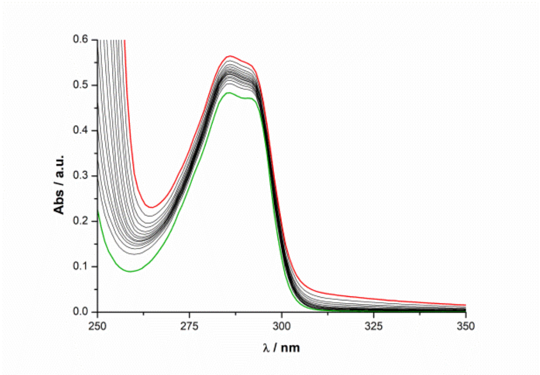

(d)

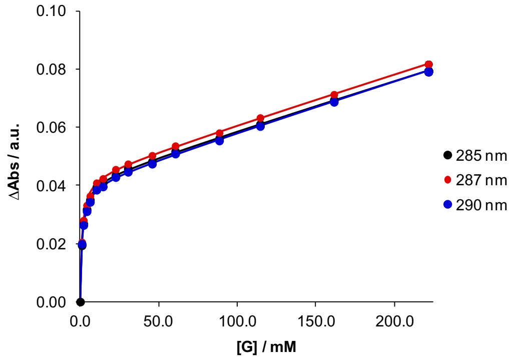

(e)

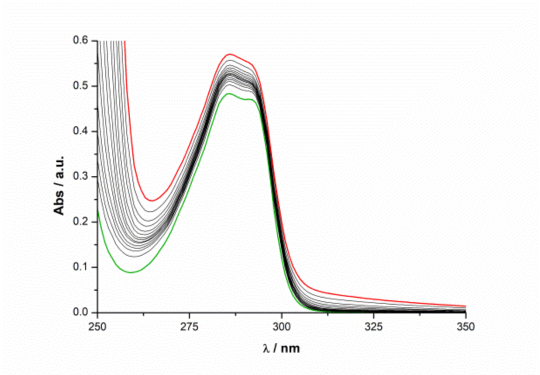

(f)

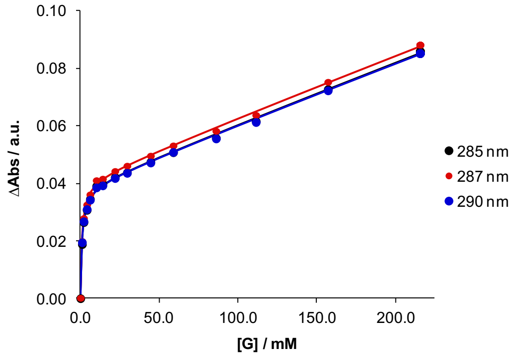

Figure S42 (a, c, e) UV-Vis spectra of **2** (0.200 mM in green) in *n*-octane at increasing concentrations of quinuclidine (from green to red), and (b, d, f) plot of the absorbance of **2** at 285 nm, 287 nm, 290 nm versus the concentration of **2** and its fittings to a 1:1+non-specific binding model.

Molecule 3

Dilution Experiment of 3 in *n*-octane

(a)

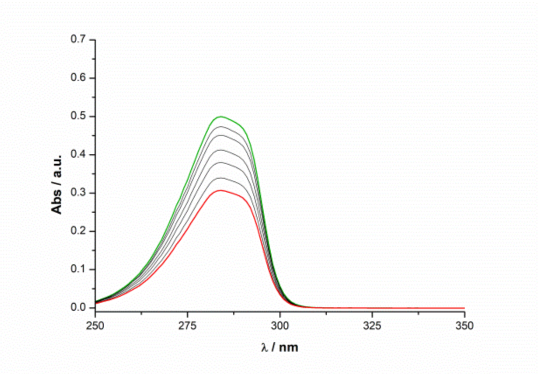

(b)

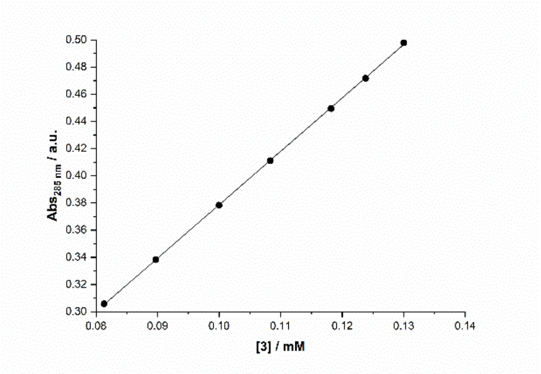

Figure S43 (a) UV-Vis spectra of **3** in *n*-octane at decreasing concentrations (from 0.130 mM in green to 0.081 mM in red), and (b) plot of the absorbance of **3** at 285 nm versus the concentration of **3** and its linear fitting.

Titrations of 3 with quinuclidine in *n*-octane

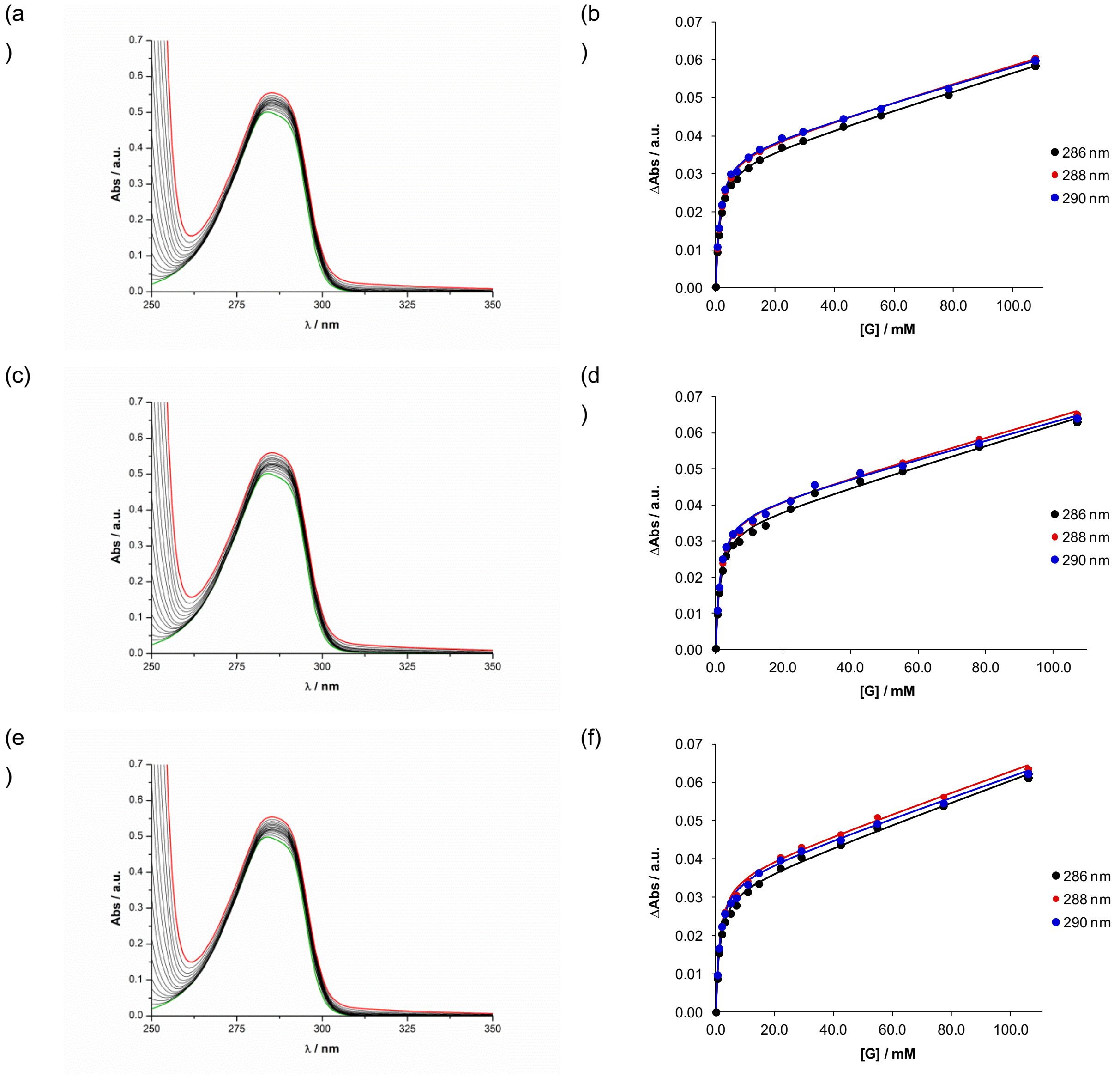

Figure S44 (a, c, e) UV-Vis spectra of **3** (0.130 mM in green) in *n*-octane at increasing concentrations of quinuclidine (from green to red), and (b, d, f) plot of the absorbance of **3** at 286 nm, 288 nm, 290 nm versus the concentration of **3** and its fittings to a 1:1+non-specific binding model.

Molecule 4

Dilution Experiment of 4 in *n*-octane

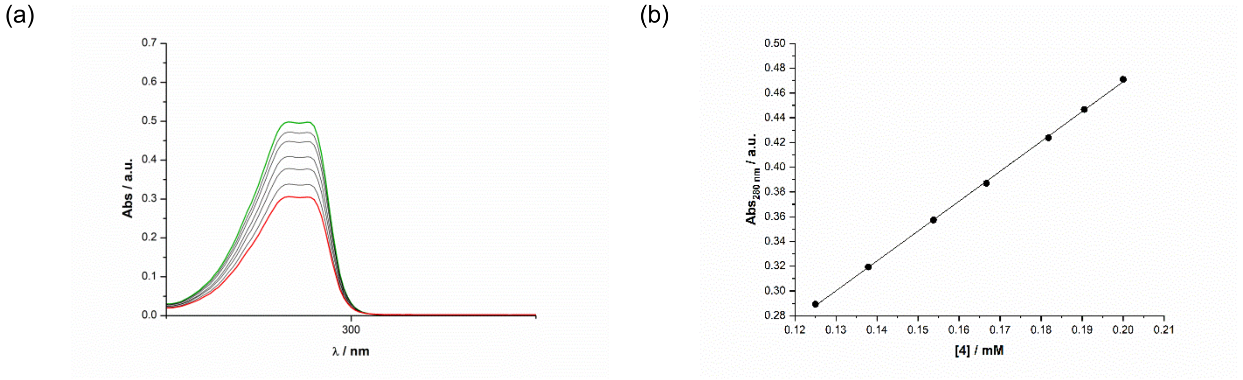

Figure S45 (a) UV-Vis spectra of **4** in *n*-octane at decreasing concentrations (from 0.200 mM in green to 0.125 mM in red), and (b) plot of the absorbance of **4** at 280 nm versus the concentration of **4** and its linear fitting.

Titrations of 4 with quinuclidine in *n*-octane

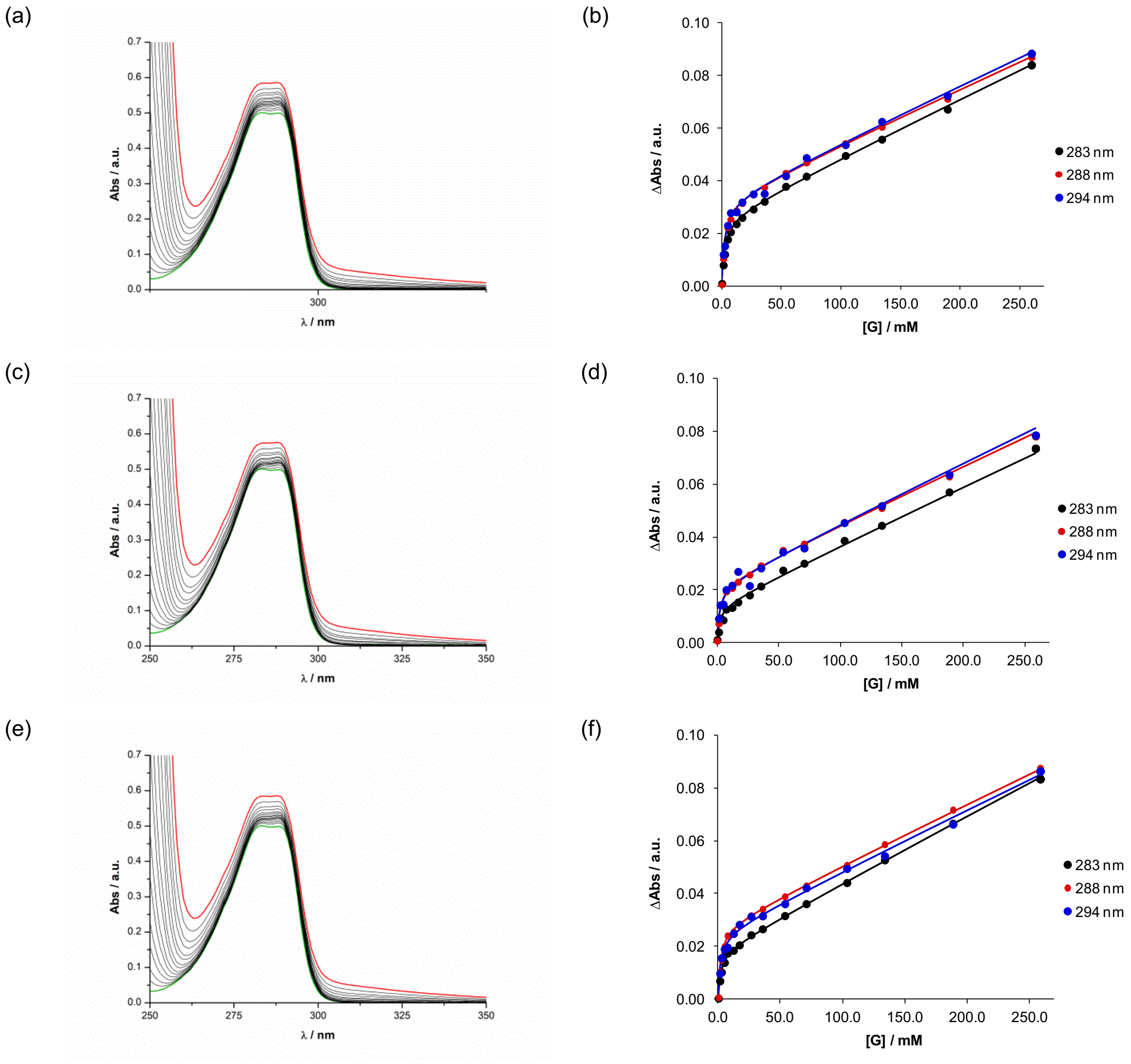

Figure S46 (a, c, e) UV-Vis spectra of **4** (0.200 mM in green) in *n*-octane at increasing concentrations of quinuclidine (from green to red), and (b, d, f) plot of the absorbance of **4** at 283 nm, 288 nm, 294 nm versus the concentration of **4** and its fittings to a 1:1+non-specific binding model.

8. References

1. Fulmer, G. R.; Miller, A. J.; Sherden, N. H.; Gottlieb, H. E.; Mudelman, A.; Stoltz, B. M.; Bercaw, J. E.; Goldberg, K. I. NMR Chemical Shifts of Trace Impurities: Common Laboratory Solvents, Organics, and Gases in Deuterated Solvents Relevant to the Organometallic Chemist. *Organometallics* **2010**, *29* (9), 2176-2179.
2. Rulev Y. A., Gugkaeva Z., Maleev V. I., North M., Belekon Y. N. Robust bifunctional aluminium-salen catalysts for the preparation of cyclic carbonates from carbon dioxide and epoxides. *Belstein J. Org. Chem.* **2015**, *11*, 1614-1623.
3. Braun, M.; Fleischer, R.; Mai, B.; Schneider, M.-A.; Lachenicht, S. The regioisomeric triphenylaminoethanols – Comparison of their efficiency in enantioselective catalysis. *Advanced Synthesis and Catalysis* **2004**, *346* (4), 474-482.
4. Sheldrick, G. M. SHELXT – Integrated Space-group and Crystal-structure Determination. *Acta Cryst. Sect. A* **2015**, *71*, 3-8.
5. Sheldrick, G. M. Crystal Structure Refinement with SHELXL. *Acta Cryst. Sect. C*, **2015**, *71*, 3-8.
6. Van de Streek, J.; Neumann, M. A. Validation of experimental molecular crystal structures with dispersion-corrected density functional theory calculations. *Acta Cryst. Sect. B*. **2010**, *66*, 544-558.
7. Macrae, C. F. ; Sovago, I. ; Cottrell, S. J. ; Galek, P. T. A. ; McCabe, P. ; Pidcock, E.; Platings, M. ; Shields, G. P.; Stevens, J. S.; Towler, M.; Wood, P. A. Mercury 4.0: from visualization to analysis, design and prediction. *J. Appl. Cryst.* **2020**, *53*, 226-235.
8. Clark, S. J.; Segall, M. D.; Pickard, C. J.; Hasnip, P. J.; Probert, M. J.; Refson, K.; Payne, M. C. First principles methods using CASTEP. *Z. Kristallogr.* **2005**, *220*, 567-570.
9. Accelrys, *Materials Studio v. 6.0*, San Diego, California, USA, 2011.
10. Perdew, J. P.; Burke, K.; Ernzerhof, M. Generalized gradient approximation made simple. *Phys. Rev. Lett.* **1996**, *77*, 3865-3868.
11. Grimme, S. Semiempirical GGA-type density functional constructed with a long-range dispersion correction. *J. Comput. Chem.* **2006**, *27*, 1787-1799.
12. Smallcombe, S. H.; Patt, S. L.; Keifer, P. A. WET solvent Suppression and Its Applications to LC NMR and High-Resolution NMR Spectroscopy. *J. Magn. Reson., Ser. A* **1995**, *117*, 295-303.
13. Hoult, D. I. Solvent Peak Saturation with Single Phase and Quadrature Fourier Transformation. *J. Magn. Reson.* **1976**, *21* (2), 337-347.

14. Anslyn, V. E.; Dougherty, D. A. *Modern Physical Organic Chemistry*; University Science Books, 2005, pp 216-221.
15. Jeener, J.; Meier, B. H.; Bachmann, P.; Ernst, R. R. Investigation of exchange processes by two-dimensional NMR spectroscopy. *J. Chem. Phys.* **1979**, *71*, 4546-4553.
16. Berger, S. Gradient-selected NOESY – A fourfold reduction of the measurement time for the NOESY experiment. *J. Magn. Reson., Ser. A* **1996**, *123*, 119-121.
